# Hybrid Nanocomposite Thin Films for Photovoltaic Applications: A Review

**DOI:** 10.3390/nano11051117

**Published:** 2021-04-26

**Authors:** Marcela Socol, Nicoleta Preda

**Affiliations:** National Institute of Materials Physics, 405A Atomistilor Street, P.O. Box MG-7, 077125 Magurele, Romania

**Keywords:** hybrid nanocomposite films, conjugated polymers, inorganic nanostructures, spin-coating, MAPLE, hybrid photovoltaic cells

## Abstract

Continuing growth in global energy consumption and the growing concerns regarding climate change and environmental pollution are the strongest drivers of renewable energy deployment. Solar energy is the most abundant and cleanest renewable energy source available. Nowadays, photovoltaic technologies can be regarded as viable pathways to provide sustainable energy generation, the achievement attained in designing nanomaterials with tunable properties and the progress made in the production processes having a major impact in their development. Solar cells involving hybrid nanocomposite layers have, lately, received extensive research attention due to the possibility to combine the advantages derived from the properties of both components: flexibility and processability from the organic part and stability and optoelectronics features from the inorganic part. Thus, this review provides a synopsis on hybrid solar cells developed in the last decade which involve composite layers deposited by spin-coating, the most used deposition method, and matrix-assisted pulsed laser evaporation, a relatively new deposition technique. The overview is focused on the hybrid nanocomposite films that can use conducting polymers and metal phthalocyanines as *p*-type materials, fullerene derivatives and non-fullerene compounds as *n*-type materials, and semiconductor nanostructures based on metal oxide, chalcogenides, and silicon. A survey regarding the influence of various factors on the hybrid solar cell efficiency is given in order to identify new strategies for enhancing the device performance in the upcoming years.

## 1. Introduction

The technological development has been the engine of the human society progress from the beginning of the “industrial revolution” in the 18th century to the present day, being in the same time responsible for the major increase in the energy consumption (anticipated to be at least double in 2100). The main concern in the energy production is represented by the enormous predominance of the non-renewable energy sources (natural gas, oil, coal) taking into account that the electricity generation involves high processing cost and the CO_2_ emissions from the burning of oil and coal leads to serious environmental issues. Naturally, the technology can have positive or negative effects on the world, the human society having the responsibility to use the technology in view of a sustainable development. Consequently, during the 20th century, numerous attempts were made to identify alternative energy sources less polluting to increase the percent of the energy supplied from renewable resources and to decrease the one obtained from non-renewable sources. Among the green and non-pollutant alternative energy sources (hydropower, wind, biomass, geothermal, and solar), the solar energy seems to be a powerful alternative to fossil fuels, the Sun giving just in one year more energy (3.8 × 10^24^ joules) than the required annual consumption (for example in 2000 was estimated as being 10,000 more) [1]. For this reason, in the last decades, the solar power was brought into the world’s spotlight emerging as the most abundant and cleanest renewable energy source available. Although the potential of sunlight energy has been available throughout history, it took centuries to develop technologies which can efficiently harvest the conversion of the radiant energy of the sun into electric energy. Thus, the solar energy can kindle the Olympic torch by concentrating the solar rays via a mirror (as in the ancient times) and can be also converted into electricity by origami-inspired large solar panel arrays disposed on the NASA space satellites [2]. In the history of the solar energy, the major breakthrough was the discovery of the photovoltaic effect in 1839 by A. E. Becquerel. The phenomenon consisting in the generation of an electric current in a material when this is exposed to sunlight is the operating principle of a solar cell. However, the modern era of the solar cells begins in 1941 when a silicon cell was described by Ohl [3], in 1954, a silicon photovoltaic (PV) cell having 6% conversion efficiency being developed by D. Chapin, C. Fuller, and G. Pearson at Bell Laboratories [4]. This PV cell was patented [5] becoming the model for the solar panels fabricated to date. Although the efficiency of the most commercial solar panels available today averages between 15% and 20% [6], the improvement of the cell efficiency will make solar energy a real alternative source in respect to fossil fuels.

**Figure 1:**
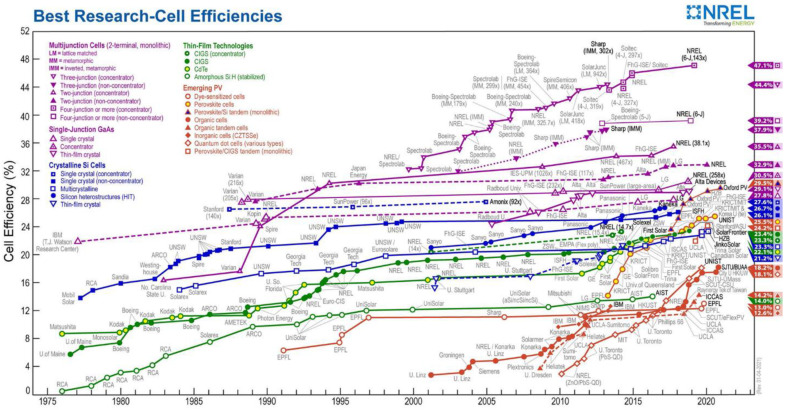
A timeline chart of the best research cell efficiencies for different photovoltaic technologies from 1976 to present according to the National Renewable Energy Laboratory (NREL) [7]. This plot is courtesy of the National Renewable Energy Laboratory, Golden, CO, USA.

Thus, an impressive development was reached in the field of the PV systems in terms of the large-scale deployment, cost reduction and performance enhancement using different materials and device architectures, in Figure 1 being given a timeline map of the best research cell efficiencies achieved by different photovoltaic technologies according to the National Renewable Energy Laboratory (NREL) [7]. 

The developed solar cell technologies can be divided into four main classes called generations [8,9,10,11] as follows: (i) The first generation based on both monocrystalline and polycrystalline silicon (Si) and on gallium arsenide (GaAs) wafers; (ii) the second generation involved thin films based on amorphous-Si, cadmium telluride (CdTe), copper indium gallium and selenium (CIGS) and cooper zinc tin sulfide solar cells (CZTS); (iii) the third generation included organic and polymeric solar cell, dye sensitized solar cells (DSSC), quantum dot solar cells, perovskite solar cells, etc.; and (iv) the fourth generation based on the composites combining organic materials (polymers, small molecules) and inorganic nanostructures, being known as “inorganic-in-organic” generation. 

Today, the PV industry is still mainly based on silicon (over 90% from the PV systems) due to the high efficiencies recorded by the PV devices manufactured with this semiconductor. Moreover, silicon presents two important advantages: (i) It is abundant in the Earth’s crust and (ii) it is stable and non-toxic [8,12]. However, a major disadvantage regarding the use of silicon in PV cells is linked to the fact that Si has a low absorption due to its indirect band gap, the fabrication of the PV systems based on this semiconductor requiring a large amount of material [8,11]. To date, the best efficiency in the PV devices involving single-junction cell based on crystalline Si is 26.7%, while an efficiency of 29.1% was achieved in the case of those based on GaAs films [13]. The fact that GaAs is a direct band gap semiconductor featured by more adequate optical properties for solar cells is mainly responsible for the higher efficiency of the PV cells based on GaAs than those employing Si. Yet, the reported values are promising taking into consideration that the efficiency of the single-junction flat solar cells can reach ~30% due to the Shockley–Queisser limit [14]. Nevertheless, involving rare materials (Ga and As), the fabrication process of GaAs solar cells is expensive [15].

During the past half century, the research was focused on developing new materials for replacing the rigid PV systems and on the lowering the processing costs of these devices for making them more accessible. Organic semiconductors are regarded as promising candidates being eco-friendly materials featured by properties, such as low processing temperatures (implying reduced fabrication costs), high absorption coefficients, appropriate mechanic flexibility, and compatibility with the plastic substrates [16,17,18,19,20,21,22], all these characteristics making them suitable for the integration in the PV devices. Furthermore, the possibility to fabricate organic devices using solution-processing techniques is very attractive from the potential commercial perspective. Thus, in 1958, Kearns evidenced the photovoltaic effect in an organic cell based on a single magnesium phthalocyanine (MgPc) layer [23]. In 1986, Tang developed an organic photovoltaic (OPV) cell in a donor/acceptor (D/A) configuration using copper phthalocyanine as donor and perylenediimide as acceptor, a power conversion efficiency of about 1% being achieved under simulated AM2 illumination [24]. In the case of the OPV devices, it was initially assumed that these cells will reach at most 10% efficiency, but in the last three decades various OPV cells based on new organic compounds and new architectures were developed in order to achieve higher efficiencies [25,26]. Hence, a 17.3% efficiency was reached for monolithic two-terminal tandem solar cells [27] while an efficiency over 18% was latter achieved for single organic layer cells [28]. These efficiency values confirm that other improvements can be made in the OPV area, one of the approaches consisting in the implementation of the bulk heterojunction (BHJ) concept [29]. In this case, the donor:acceptor (D:A) components are mixed in solution and deposited as a single film. The BHJ cell architecture presents the following major advantage: the exciton dissociation efficiency and the charge transport efficiency are improved due to the formation of a larger interface between the constituent materials in comparison with other cell architectures [10,20,24].

**Figure 2:**
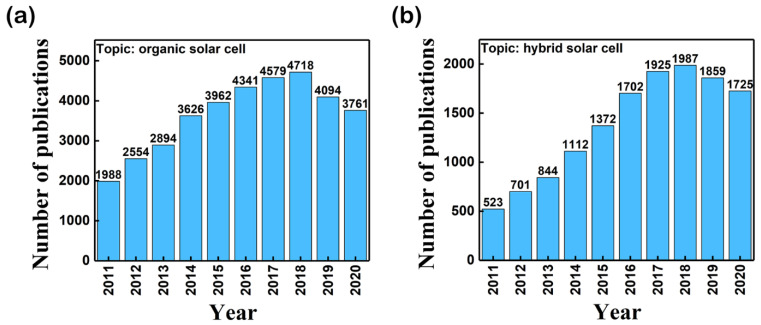
The number of the scientific publications referring to the topic (**a**) “organic solar cell” and (**b**)“hybrid solar cell” published between 2011 and 2020 (source: web of science [37]).

Among the most important parameters that can dramatically influence the efficiency of the OPV cell structures are the low carrier mobility (10^−4^ cm^2^V^−1^s^−1^) of the organic semiconductors and the charge carrier transport which is related to the presence of the traps in the organic films [30,31]. The carrier mobility of the organics, much lower than that of the inorganic materials (for example the electron mobility at room temperature of pure Si is 1400 cm^2^V^−1^s^−1^ [32]) is linked to the fact that hopping is the dominant charge transport mechanism in organics instead of band transport in inorganics [33]. Recently, various studies report that the PV cell efficiency can be enhanced by involving composite films relatively easily obtained by embedding inorganic semiconductors nanostructures (materials featured by high intrinsic carrier mobility and thermal stability) in an organic compound [8,34,35,36]. In this context, the BHJ concept can be extended at inorganic:organic layers designed for fabricating hybrid photovoltaic (HPV) cell structures with improved parameters like open circuit voltage (V_OC_), short-circuit current density (J_SC_), maximum power (P_max_), fill factor (FF), and power conversion efficiency (PCE) [35]. The interest for the solar cell research area is emphasized by the trend in the number of the scientific articles published in the last 10 years having as topic “organic solar cell” or “hybrid solar cell” presented in Figure 2. 

A HPV cell structure contains active layers based on stacked (inorganic/organic) films or on blends (inorganic:organic) films involving one or two organic materials and inorganic nanostructures. Moreover, supplementary inorganic:organic composite layers (also known as buffer layers), such as the electron transport layer (ETL) or hole transport layer (HTL), can be used beside the organic blend (active layer) for improving the cell efficiency. Generally, inorganic nanostructures based on semiconductors (Si, chalcogenides, metal oxides, etc.) or metals (Au, Ag, Cu, etc.) are used in the preparation of the hybrid layers. 

**Figure 3:**
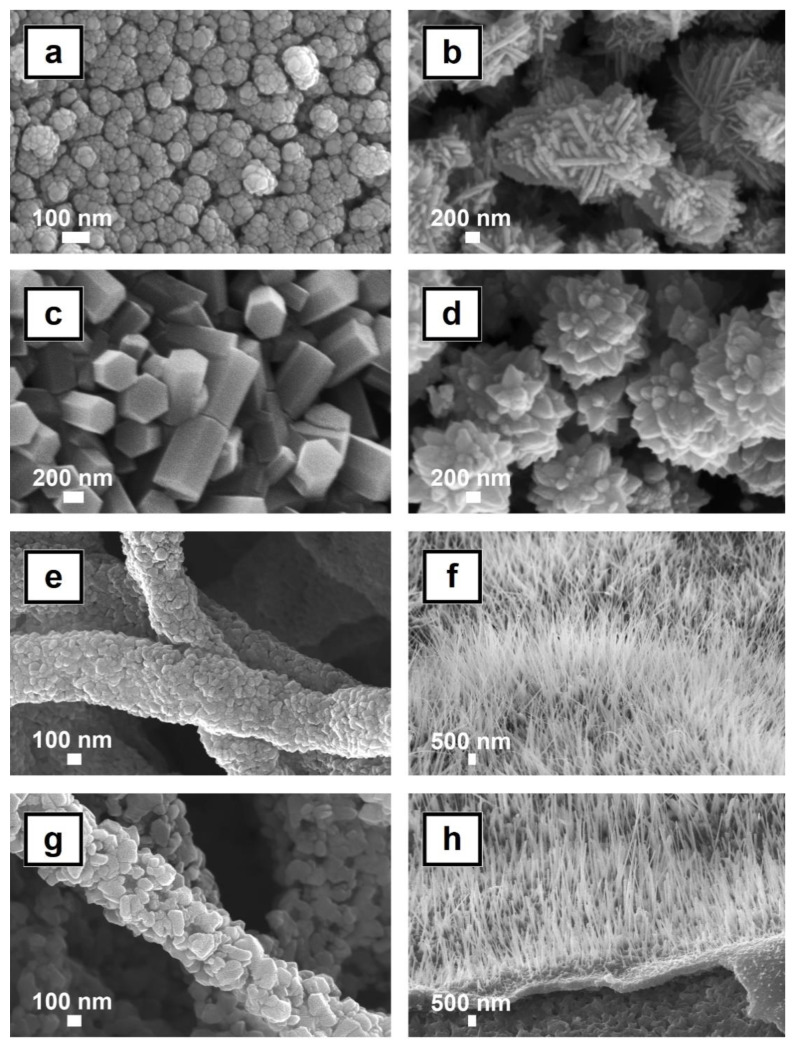
SEM images of ZnO (**a**–**f**) and CuO (**g**,**h**) nanostructures with various morphologies prepared by wet and dry techniques. (**a**) reprinted with permission from [44]. Copyright 2020 Elsevier. (**b**,**d**) reprinted with permission from [45]. (**c**) reprinted with permission from [46]. Copyright 2013 Elsevier. (**e**,**g**) reprinted with permission from [47]. Copyright 2020 Elsevier. (**f**) reprinted with permission from [48]. Copyright 2016 Elsevier. (**g**) reprinted with permission from [49]. Copyright 2015 AIP Publishing.

Among the metal oxides nanostructures, those based on ZnO, CuO, and TiO_2_ are the most used in the PV area. These three environmentally friendly materials are featured by morphologically rich families of nanostructures (spheres, wires, rods, tubes, tetrapods, needles, spindle-shaped platelets, etc.) that can be relatively easily obtained by numerous wet and dry techniques [38,39,40,41,42,43,44,45,46,47,48,49]. Figure 3 illustrates some nanostructures based on ZnO or CuO with different morphologies (particles, prisms, flowers, flakes, fibers and wires) prepared by straightforward paths such as chemical precipitation [44,45], electroless deposition [46], biomorphic mineralization [47], and thermal oxidation in air [48,49]. 

From the chalcogenides nanostructures, those based on Cd (CdS, CdSe, CdTe) and Pb (PbS, PbSe) gained a significant in the PV field being used as photovoltaic absorbers and the potential of those based on others compounds, such as SnS, FeS_2_, CuInS_2_, CuInSe_2_, etc., was also evaluated [50]. 

Metal nanostructures synthesized in different morphologies, sizes and compositions by many chemical approaches can be also integrated in the HPV cell structures [51,52,53,54]. The addition of the metal nanostructures can enhance the carrier mobility and the local absorption in the active layer due to the localized surface plasmon resonance (LSPR) effect leading to the improvement of the performances of the organic cells [54,55].

It is well known that the features like thickness, homogeneity, transmittance, etc., of the layers involved in the development of a PV cell structure are strongly related to the experimental parameters characteristics to each technique employed in their fabrication. From this perspective, the deposition method must be adequately chosen in order to obtain films with suitable properties. 

Solution-processing techniques, spin-coating, inkjet printing and doctor blading are widely used in the fabrication of the OPV cells. Although the inkjet printing is a non-contact process that uses a small quantity of materials and doctor blading is a precision coating technique that allows the deposition of a well-defined film thickness [56], spin-coating is still the most common path in the deposition of the organic active layer for the OPV structures [57] and of the hybrid active layer for the HPV devices [8,36]. The relatively inexpensive method, spin-coating allows a quick and easy deposition of the layers involving the following steps: deposition of the mixture solution on the substrate, substrate spinning, solvent evaporation and formation of the thin film. The thickness of the films is strongly influenced by the last two steps, which are usually overlapped [58]. Thus, the organic compound is firstly dissolved in a proper solvent (typically 10 mg/mL [36]), the obtained solution being deposited on a rigid substrate with a dosing unit (dispenser) or manually with a syringe. The solution can be deposited on the substrate in a dynamic or a static regime. In the dynamic regime, the solution is deposited on a substrate rotated at a low speed. This approach is more appropriate when the fluid or the substrate present poor wetting abilities, the formation of discontinuities inside the film being avoided. In the static regime, the solution is deposited on the substrate before starting the centrifugation. The quantity of the solution deposited on the substrate is chosen related to its viscosity and to the substrate dimensions. Further, the substrate is continuously rotated at a high speed (up to 10,000 rot/min) in order to spread the solution due to the rotational movement and to evaporate the solvent for obtaining the thin film. The frequency depends on the substrate and fluid properties while the rotation time varies between few seconds until minutes depending on the film thickness intended to be obtain. The properties of the deposited film are strongly related to the thickness of the layer which, in turn, depends on the nature of the spread solution (viscosity, surface tension, drying rate, etc.) and on the parameters of the spinning process (rotation speed and rotation time). 

Laser-processing technique, i.e., Matrix-Assisted Pulsed Laser Evaporation (MAPLE) is a relatively new method being developed in the late 1990s by the US Naval Research Laboratory for processing organic compounds (especially polymers) layers with the preservation of their chemical structure during the deposition [59]. Thus, using small quantities of material (typically below 5 wt%), MAPLE allows the deposition of organic or hybrid layers for OPV [60,61,62,63] or HPV [44,64,65], respectively. Compared to the pulsed laser deposition (PLD), a technique employing a solid target obtained from pressed powder or pellets, MAPLE involves a frozen target prepared from the material that is intended to be deposit as thin film and a suitable solvent, used as matrix [66,67]. Hence, the selected solvent must meet two requirements: To be a good solvent for the organic material and to absorb at the laser wavelength used in the deposition process. Additionally, in comparison with the PLD where the polymer chains can be broken due to the higher laser fluences, the MAPLE requires lower fluences (below 0.5 J/cm^2^) for preventing the damage of the chemical structure of the “soft” organics (small molecules, polymers, biomaterials, etc.) used as raw materials [67]. The MAPLE deposition involves the following steps: (i) firstly, the organic material is dissolved in the appropriate solvent, the solution being stirred for homogenization; (ii) the organic solution is frozen in the liquid nitrogen in order to obtain the solid frozen target used in the deposition; and (iii) during the MAPLE process, when the laser pulses impinged the target, the energy absorbed by the matrix is converted in thermal energy, the solvent and organic molecules being ejected simultaneously from the target: the volatile solvent molecules are pumped away by the vacuum system, whereas the organic molecules form the layer on the surface of the substrate [65,67]. It is worth mentioning that during the MAPLE process, organic and solvent clusters can be ejected toward the substrate resulting in the formation of droplets or aggregates structures on the surface of the deposited films, their presence influencing the contact between the layers from the developed structure [68]. Nevertheless, in the MAPLE deposition, an accurate control of the thin film thickness can be achieved by tuning the experimental parameters such as laser fluence, repetition rate, target-substrate distance, temperature of the substrate and number of the applied laser pulses. Although UV lasers are usually employed in the MAPLE process, other MAPLE-based techniques, such as resonant infrared-MAPLE (RIR-MAPLE) or emulsion-based RIR-MAPLE using IR pulses, were developed for the deposition involving organic solvents characterized by vibrational frequencies in the infrared region [69]. Additionally, combinatorial-MAPLE (C-MAPLE) is an alternative technique used to grow, in a single-step process, thin hybrid bio-coatings with a gradient of composition, as was reviewed in [68]. 

Although spin-coating is a simple and low-cost solution deposition technique, it has some disadvantages: (i) Only 2–5% from the dispersed solution is used to form the film; (ii) stacked multilayer structures can be prepared using only orthogonal solvents; and (iii) difficulty in an accurate control of some features such as homogeneity, roughness, etc., of the obtained films. By comparison, MAPLE is a relatively high-cost technique mainly due to the special required equipment, featured by the following major advantages: (i) The ability to prepare multilayers using the same solvent, the deposition of the second layer taking place without affecting the first deposited layer [61]; (ii) accurate thickness control of the homogeneous and adherent films over desired substrate; and (iii) the ability to deposit ultra-thin films. It must be highlighted that thin films can be deposited by MAPLE even on plastics [60,70], the deposition on such substrates being very useful in flexible electronics, an emerging technology area which lately attracted the research attention due its potential applications in PV devices, aerospace, bio-medicine, etc.

Albeit the present work is focused on the applications of the hybrid composite layers in the PV area, it is worth mentioning that such layers obtained by spin-coating and MAPLE techniques are also applied in other fields. In the following are given some examples of layers deposited by spin-coating (i–v) or by MAPLE (vi–x): (i) Films based on poly(vinylidene fluoride) and strontium bismuth tantalite (SBT) nanoparticles for ferroelectric devices [71]; (ii) films based on poly(vinylidene fluoride) and Ni_0.5_Zn_0.5_Fe_2_O_4_ nanoparticles for magnetoelectric devices [72]; (iii) films based on poly(vinylidene difluoride) and Fe_3_O_4_ nanoparticles for battery electrodes [73]; (iv) films based on poly(methyl methacrylate) and WS_2_ nanosheets for nonlinear optic [74]; (v) films based on poly(ε-caprolactone) and various materials such as Al_2_O_3_, graphene, carbonated hydroxyapatite or TiO_2_ for tissue engineering [75]; (vi) films based on poly(lactic acid), poly(vinyl alcohol), chitosan, eugenol, and Fe_3_O_4_ nanospheres for antimicrobial coatings [76]; (vii) films based on cyclodextrin, cefepime and ZnO nanoparticles [77] for bioactive coatings; (viii) films based on poly(ethylene glycol) and ZnO nanoparticles for antimicrobial surfaces [78]; (ix) films based on lanthanide-doped upconversion nanoparticles (NaGdF_4_: Yb^3+^, Er^3+^) with or without immunoglobulin G (IgG) for biological devices in tissue engineering and tissue regeneration [79]; and (x) films based on poly(ethylene glycol)-block-poly(ε-caprolactone) methyl ether copolymer, lactoferrin and hydroxyapatite for bioactive surfaces for bone regeneration [80]. 

Concerning the scalability of the spin-coating and MAPLE techniques, it must be emphasized that both are suitable tools for layer deposition on small area, being very useful for developing PV cells at laboratory scale. However, for transferring the most efficient PV cells fabricated by these two processing methods to techniques compatible to large-area solar cells it is important to identify and understand the influence of various experimental parameters and material type on the electrical performance of the developed solar cells. Thus, such information can be considered useful guidelines for achieving an efficient transfer from small-area PV cell technology to large-area PV cell fabrication.

Lately, various papers have summarized the achievements attained in the field of the PV cells fabricated by various processing techniques (spin-coating, blade coating, spray coating, vacuum evaporation, screen printing, inkjet printing, etc.) [8,11,34,35,36,81,82,83,84,85]. However, a review focused on the development achieved in the PV cells based on composite layers involving inorganic semiconductors nanostructures embedded in the organic materials can be very helpful in understanding the impact of different factors on the device performance and finding new approaches to overcome the current limitations in the upcoming years.

In this context, the present work reviews recent progress in the field of the photovoltaic cells based on hybrid nanocomposite thin films. Thus, comprehensive but non exhaustive, this overview (i) summarizes the organic compounds (conducting polymers and metal phthalocyanines as *p*-type materials and fullerene derivatives and non-fullerene compounds as *n*-type materials) and inorganic nanostructures (semiconductors, such as metal oxide, chalcogenides, and silicon) which are frequently used in the preparation of the organic:inorganic mixtures for the deposition of hybrid layers; (ii) discusses the PV cells developed with such hybrid composite thin films deposited by spin-coating (the most used deposition technique) and MAPLE (a relatively new deposition technique); and (iii) explores their future perspectives. 

## 2. Organic Compounds and Inorganic Nanostructures—Materials for Designing PV Cells

Material selection for the active films, buffer layers, and electrodes plays a key role in the development of the PV cell structures. Moreover, the device architecture is one of the most important parameters for evaluating the performance of PV cells. The device architecture can be classified as regular (also known as conventional) and inverted depending on which transport material (electron or hole) encountered first the incident light. Thus, in a regular device architecture, indium tin oxide (ITO) is the transparent conductive oxide (TCO) most used as anode due to its high work function, increased transparency and reduced electrical resistivity [86], while aluminum is commonly used as the cathode being featured by a low work function [35,87]. Lately, other TCOs, such as fluorine-doped tin oxide (FTO) or aluminum-doped zinc oxide (AZO), were also involved in the fabrication of both OPV and HPV (containing an organic:inorganic composite layer) devices [88,89]. In an inverted device architecture, a configuration in which the charge collection is reversed in comparison with the regular configuration meaning that the electrons are collected by the TCO front electrode (*n*-type materials such as ITO, FTO, TiO_2_) and the holes by the metal back contact, the transparent electrode working as cathode and the metal having a high work function (gold or silver) working as the anode [90]. 

Important components of the PV cells, the buffer transport layers, HTL (also known as anode buffer layer (ABL) or electron blocking layer (EBL)) and ETL (also known as cathode buffer layer (CBL) or hole blocking layer (HBL)) can improve the device performances by reducing the recombination for one type of charge and increasing for the other [91]. Usually, poly(3,4-ethylenedioxythiophene): poly(styrene sulfonate) (PEDOT:PSS), a conducting polymer characterized by a high optical transparency in the visible domain, a high work function (relative to ITO), a high hole conductivity, and a good chemical stability in air [92] is used as HTL in the OPV and HPV structures. Recently, the potential of metal oxides like molybdenum trioxide (MoO_3_) or tungsten trioxide (WO_3_) as HTL was also evaluated [93,94,95]. However, *n*-type metal oxides such as ZnO and TiO_2_ are frequently used as ETL. Thus, the energy levels of ZnO and its high electron mobility prevents the charge recombination [90]. In addition to its hole blocking ability, ZnO can also act as a barrier against the UV light avoiding the photodegradation of the organic active film [90]. Various studies show that the use of ABL or CBL layers containing organic:inorganic composite can also improve the PV device performance [96,97]. 

Some of the OPV and HPV device architectures currently developed are illustrated in Table 1 being exemplified with the organic compounds and inorganic semiconductor nanostructures involved in their fabrication according to the data collected from the literature review [94,96,97,98,99,100,101,102,103,104].

Next, a survey regarding the organic compounds (Section 2.1.) and inorganic nanostructures (Section 2.2) that are commonly used in the active layers of the PV cells and some of their properties will be provided. 

### 2.1. Organic Compounds for PV Applications

Usually, the active layer of a PV cell based on BHJ consists in a blend formed by a *p*-type organic semiconductor as electron donor and a *n*-type organic semiconductor as electron acceptor. The blends can be formed by two or more organic (polymers, small molecules, etc.) components. The design of organic compounds characterized by higher solubility and suitable band gap and energy levels in order to increase the V_OC_ and J_SC_ values is a permanent demand taking into account that the optical and electrical properties of the organic materials used in the active layer are mainly responsible for the device parameters [105]. For instance, the donor materials play the main role in the light harvesting in the visible range even if the acceptor materials can also contribute to this process. Thus, organic semiconductors characterized by a high absorptivity, a band gap value lower than 2.0 eV and by a deep HOMO energy level (lower than the threshold for air oxidation, around −5.2 eV [106]) are preferred as donors for ensuring the air stability [107]. For this reason, many papers have focused lately on the synthesis of such organic semiconductor materials featured by a low band gap [108].

#### 2.1.1. Conducting Polymers and Metal Phthalocyanines as *p*-Type Materials in the PV Cells

Poly(3-hexylthiophene-2,5-diyl) (P3HT) is probably the most studied conjugated polymer in the organic solar cell area, the research carried on this inexpensive poly(alkylthiophene) having significant consequences in the development of the OPV devices. P3HT is featured by a band gap of ~1.9 eV, a HOMO energy level at 5.2 eV and a high hole mobility [109]. In the OPV and HPV cells, P3HT is extensively used as a *p*-type material in pair with fullerenes, fullerene derivatives, or others *n*-type materials [110,111]. One of the main advantages of P3HT is the fact that it allows low temperature thin film processing from solutions [87,112]. However, due to its band gap value, P3HT can harvest only in a narrow range of the solar spectrum (350–650 nm range). This is one of the main factors that limits the efficiency of the PV devices based on P3HT being known that the maximum photon flux of the solar spectrum is around 1.6–1.8 eV [113]. Therefore, in order to improve the performance of the PV cells, many efforts have been carried to design new compounds with a lower band gap.

During the last years, donor-acceptor (D-A) polymers, materials with narrow band gap obtained by alternating electron-rich units (donor) and electron-deficient units (acceptor) covalently bonded within the same chain have been developed by different research groups [114,115,116,117]. Poly [[4,8-bis[(2-ethylhexyl)oxy]benzo[1,2-b:4,5-b’]dithiophene-2,6-diyl][3-fluoro-2-[(2-ethylhexyl)carbonyl]thieno[3,4-b]thiophenediyl]] (PTB7) is a polymer featured by a band gap of ~1.6 eV and a good hole mobility that can be relatively easily deposited from solutions being soluble in various organic solvents [118]. A dithienyl derivative of PTB7, poly[4,8-bis(5-(2-ethylhexyl)thiophen-2-yl)benzo[1,2-b;4,5-b’]dithiophene-2,6-diyl-alt-(4-(2-ethylhexyl)-3-fluorothieno[3,4-b]thiophene-)-2-carboxylate-2-6-diyl)] (PTB7-Th, also known as PCE10, PBDTTT-EFT, PBDTT-FTTE) is featured by an extended absorption due to its band gap of ~1.58 eV and a favorable HOMO energy level at 5.2 eV [119]. Thus, OPV and HPV devices with high efficiency were fabricated involving PTB7 [120,121] and PTB7-Th [122,123]. For example, for PV cells based on blends containing PTB7-Th and *n*-type materials such as [6,6]-phenyl C71-butyric acid methyl ester (PC71BM) [118] or 3,9-bis(2-methylene-(3-(1,1-dicyanomethylene)-indanone))-5,5,11,11-tetrakis(4-hexylphenyl)-dithieno[2,3-d:2′,3′-d’]-s-indaceno[1,2-b:5,6-b’]dithiophene (ITIC) [123] high efficiency values of 10.8% and 10.42%, respectively, were achieved.

Poly[2,6-(4,4-bis-(2-ethylhexyl)-4H-cyclopenta[2,1-b;3,4-b′]dithiophene)-alt-4,7(2,1,3-benzothiadiazole)] (PCPDTBT, also known as C-PCPDTBT) or its derivative poly[(4,4-bis(2-ethylhexyl)-dithieno[3,2-b:2′,3′-d]silole)-2,6-diyl-alt-(2,1,3-benzothiadiazole)-4,7-diyl] (PSBTBT, also known as Si-PCPDTBT) are other polymers characterized by a low band gap of ~1.5 eV and very good charge transport properties, both features being essential in the fabrication of the PV devices with high efficiency [124,125].

Poly[N-9′-heptadecanyl-2,7-carbazole-alt-5,5-(4′,7′-di-2-thienyl-2′,1′,3′-benzothiadiazole)] (PCDTBT) is a polycarbazole-based conjugated polymer having a band gap of ~1.8 eV, a deep HOMO energy level at 5.45 eV and a good stability which was also successfully used in the OPV area [107].

The potential application of other conjugated polymers such as poly(phenylenevinylene) (PPV) and especially its derivatives, poly[2-methoxy-5-(2-ethylhexyloxy)-1,4-phenylenevinylene] (MEH-PPV) and poly[2-methoxy-5-(3′,7′-dimethyloctyloxy)-1,4-phenylenevinylene] (MDMO-PPV) in the PV domain was also evaluated [126]. Featured by a large band gap of ~2.4 eV [127], PPV can be deposited from solutions as thin films [128]. Thus, hybrid thin films based on functionalized PPV (amines or pentafluorophenyl esters as side chains) and inorganic nanocrystals were fabricated by a layer-by-layer approach and their photovoltaic properties were investigated [129]. MEH-PPV characterized by a band gap of ~2.2 eV [130], a HOMO energy level at 5.3 eV [126] and a high hole mobility [131] and MDMO-PPV featured by a band gap of ~2.2 eV ([132] are the PPV derivate most commonly integrated in the OPV and HPV cells [132,133].

Originally known as organic dyes and pigments, metal phthalocyanines are small molecules that are integrated in the OPV field since 1986 (Tang 1986) due to their specific properties, such as high absorption coefficients (~10^5^ cm^−1^), and high thermal and chemical stability [134,135,136]. These organic compounds found also applications in the organic field effect transistor (OFET) and organic light emitting devices (OLED) area [137]. As active layers in the PV cells, copper phthalocyanine (CuPc), and zinc phthalocyanine (ZnPc) characterized by a high hole mobility (compared with other organic semiconductors) and a long exciton diffusion length (~15 nm for CuPc and ~30 nm for ZnPc) can be used either as blends (with other organic compounds) or as composites (with inorganic nanostructures) [44,138,139]. Metal phthalocyanines can be directly deposited as thin films by vacuum evaporation or by solvent evaporation, being organic compounds soluble in a wide variety of solvents [140,141,142]. In the PV devices, metal phthalocyanines are frequently used for their light absorption with acceptor materials (e.g., C_60_ fullerene) or as additional *p*-type material for enhancing the light absorption of other *p*-type material (e.g., P3HT) [143]. In the hybrid solar cells, ZnPc or CuPc are paired with inorganic nanostructures, like ZnO, TiO_2_, etc. Thus, an enhancement in the photovoltaic response was reached for the cell based on hybrid layers containing CuPc and ZnO nanoparticles (V_OC_ = 0.87 V and J_SC_ = 3.6 × 10^−5^ A) in comparison with the cell based only on a CuPc layer (V_OC_ = 0.57 V and J_SC_ = 1.37 × 10^−7^ A) [141]. Additionally, adding CuPc (5%) in a composite based on P3HT and TiO_2_, the absorption of the active layer is increased, the photoluminescence intensity being decreased [144]. A conversion efficiency of 0.53% is achieved for a cell based on CuPc:C60 and ZnO nanowire arrays whereas this parameter is only 0.13% for the cell without inorganic nanostructures [145]. A 2.8% efficiency was obtained for a hybrid solar cell containing a ZnO nanorods layer on which ZnPc:C_60_ active layer was deposited by evaporation [146]. Moreover, metal phthalocyanines can be integrated as the charge selective layers or added into the photoactive layer in order to improve the absorption and the stability of the perovskite solar cells [147].

#### 2.1.2. Fullerene Derivatives and Non-Fullerene Compounds as *n*-Type Materials in the PV Cells

Fullerene derivatives are widely applied as acceptors materials in the PV area, but recently other organic semiconductors featured by a high electron affinity, high charge carrier mobility, and proper miscibility with the donor material were also taken into consideration [148]. Although, fullerene C_60_ has a relatively high electron mobility (up to 1 cm^2^/Vs), a very useful feature for its integration in the OPV or HPV devices based on stacked layers or BHJ [149], the low solubility of C60 hinders its deposition from solutions as thin films. For this reason, many attempts were carried out for replacing fullerene C_60_ with other fullerene derivative compounds with a better solubility which can allow the deposition of both *n*-type and *p*-type materials from solution.

[6,6]-phenyl-C61-butyric acid methyl ester (PCBM, also known as PC60BM, PC61BM) is often used as acceptor material in the fabrication of the solar cell structures due to its high electron mobility, high electron affinity, and good solubility in various organic solvents [150]. In addition, PCBM derivatives compounds recently prepared are promising new acceptors being characterized by absorption in the visible spectral domain and appropriate LUMO energy level [87,150].

[6,6]-phenyl-C71-butyric acid methyl ester (PC71BM) (also known as PC70BM) presents improved absorption properties in the visible region (relative to PCBM) taking into account that the energetic transitions forbidden in C60 are allowed in the non-symmetrical C70 cage [151]. Therefore, PC71BM was successfully integrated in the PV devices with high efficiencies [96,152].

The non-fullerene acceptor materials are an attractive alternative for replacing fullerene derivatives (even for the flexible electronics) since properties such as absorption, energy levels and device stability can be enhanced, modified or tuned by molecular design [119,153]. Among these compounds, 3,9-bis(2-methylene-(3-(1,1-dicyanomethylene)-indanone))-5,5,11,11-tetrakis(4-hexylphenyl)-dithieno[2,3-d:2′,3′-d’]-s-indaceno[1,2-b:5,6-b’]dithiophene (ITIC) exhibits strong and broad absorption, suitable energy levels, high electron mobility and a good miscibility with various donor polymers [154]. ITIC is featured by a push–pull architecture (an electron-rich central unit with four or more aromatic fused rings flanked by two electron-deficient units forming an A–D–A molecular structure) which facilitates the intramolecular charge transfer and improves the light absorption by broadening the absorption spectrum [119,154]. Thus, ITIC was used in combination with conjugated polymers (P3HT, PTBT-Th) and inorganic nanostructures (ZnO nanoparticles) for developing HPV devices with improved efficiencies [96].

### 2.2. Inorganic Semiconductor Nanostructures for PV Applications

Nowadays inorganic nanostructures with tailored size and morphology are frequently used as components of the hybrid layers for various PV cell configuration, the nanostructures based on inorganic semiconductors, such as ZnO, CuO, TiO_2_, CdS, PbS, Si, etc. [34,35] being usually embedded in the organic active layer or in the supplementary layer. Hence, the semiconductor nanostructures can be firstly obtained by various chemical preparation routes (precipitation, hydrothermal, solvothermal, etc.) and then added in the solution containing the organic compound. Moreover, the nanostructures can be synthesized in an organic media that can act as solvent for the organic compounds allowing the formation of a blend that further is deposited as bulk-heterojunction [155].

Among the metal oxides, ZnO, CuO, and TiO_2_ occupy a special place being technological key materials due to their interesting characteristics which includes: (i) Relatively easy synthesis by solution-based chemical approach employing inexpensive equipment; (ii) involvement of low-cost and easily accessible chemical reagents in their chemical synthesis allowing the preparation of large metal oxide amounts; (iii) minimal environmental impact; (iv) excellent mechanical, thermal and chemical stability; (v) specific optical and electrical properties that can be easily tuned during the preparation stage in order to be suitable for a wide range of applications [38,40,41,43,156,157]. ZnO and TiO_2_ are *n*-type metal oxides with a wide band gap (~3.3 eV for ZnO [38], ~3.2 eV for TiO_2_ atanase, and ~3.0 eV for TiO_2_ rutile [41,158]) while CuO is a *p*-type metal oxide with a narrow band gap ~1.2 eV [40,159] (all band gap values are given for the metal oxides in bulk form). ZnO and TiO_2_ are used as acceptors in combination with polymer donors in the PV devices due to their high electron mobility and high transparency [35,94] while CuO is suitable for integration in the solar cells because it assures a better light harvesting. Thus, studies carried out on HPV structures involving TiO_2_ as nanorods array layer or as composite with organic semiconductors (P3HT:PCBM) revealed that the presence of metal oxide nanostructures improved the performance of the PV devices by providing a direct path for the charge transport [42,160,161].

Colloidal semiconductor nanocrystals (also known as quantum dots (QDs)) based on metal chalcogenide such as Cd-compounds (CdS, CdSe, CdTe), Pb-compounds (PbSe, PbTe, PbS), ternary chalcogenides (CuInS_2_, CuInSe_2_) or other semiconductor compounds (PbI_2_, FeS_2_, etc.) can also be mixed with different organic semiconductors for developing hybrid layers for PV applications [50,162]. The growing interest shown to the colloidal semiconductor nanomaterials over the past decade is related to (i) their unique size-dependent optoelectronic properties (based on the quantum confinement effects), (ii) the relatively simple solution-based synthesis used in their preparation, and (iii) the great potential in the development of low-cost, low-energy consumption and flexible optoelectronic device due to their solution-processed characteristic. Accordingly, by controlling the experimental parameters involved in their chemical synthesis (precursor type, ligand type, reactants concentration, temperature, time, etc.), an accurate control over size and size distribution, shape, crystal structure, composition and coating ligand of the prepared colloidal nanocrystals can be achieved [163]. Furthermore, semiconductor nanocrystals can be regarded as promising substitutes for the fullerene acceptors due to their features, such as (i) tuning of the band gap and energy level by the quantum confinement effect (linked to the colloid properties); (ii) strong and broad absorption at energies higher than the band edge; (iii) high dielectric constant useful for overcoming the strong exciton binding energy of the conjugated polymers and (iv) electron mobility higher compared to the organic materials. Therefore, for fabricating highly efficient PV devices involving such colloid nanostructures, many efforts have been made for obtaining a continuous percolation network between the colloidal chalcogenides nanocrystals and the organic compound taking into account that the polymer:nanostructures contact area can be influenced by the presence of the surface traps that further can have a strong impact on the charge generation and transport [50,164].

Colloidal nanostructures based on Cd-chalcogenides are promising photovoltaic materials due to their adequate band gaps and high absorption. CdS has a band gap of ~2.42 eV [165], well suited to the visible light component of solar energy, its colloidal nanostructures being applied in various fields like solar cells, light emitting diodes or photodetectors [166]. CdSe is characterized by a band gap of ~1.74 eV [167], its colloidal nanostructures (relatively easily synthesized) being especially used in combination with thiophene-based polymers (e.g., P3HT [50]) in the PV cells taking into account that CdSe is an electron acceptor which presents an absorption in the visible part of the spectrum. CdTe is featured by a bad gap of ~1.54 eV [168], its colloidal nanostructures being usually prepared by aqueous solution-based approaches, more environmentally friendly than oil-based pathways generally used in the synthesis of the CdSe colloidal nanostructures [169].

Colloidal nanostructures based on Pb-chalcogenide are preferred in the PV field due to their photosensitivity in the near-infrared (NIR) region and to the possibility to tune their band gap from 0.3 eV to >1.5 eV during the synthesis route [170]. Hence, solar cell structures based on active layers embedding PbS or PbSe colloidal nanostructures (the layers being deposited from solution) revealed improved efficiencies and cell stability relative to the cells prepared in the absence of the semiconductor nanostructures [171].

Colloidal nanostructures based on ternary chalcogenides such as CuInS_2_ or CuInSe_2_ are highly attractive for the PV field because they contain less-toxic materials and exhibit adequate optical and electrical properties [172].

Colloidal nanostructures of other semiconductors like PbI_2_ and FeS_2_ can be also integrated in the PV cells improving the absorption properties of the active layer [173].

The performance of the PV devices can be also enhanced by embedding silicon nanostructures (nanowires or nanoparticles) within the active organic material, these providing a large surface area and a direct path for charge transport [174].

## 3. Spin-Coating and Matrix-Assisted Pulsed Laser Evaporation (MAPLE)—Techniques Used in the Deposition of the Hybrid Nanocomposites Thin Films for Developing PV Cells

**Figure 4:**
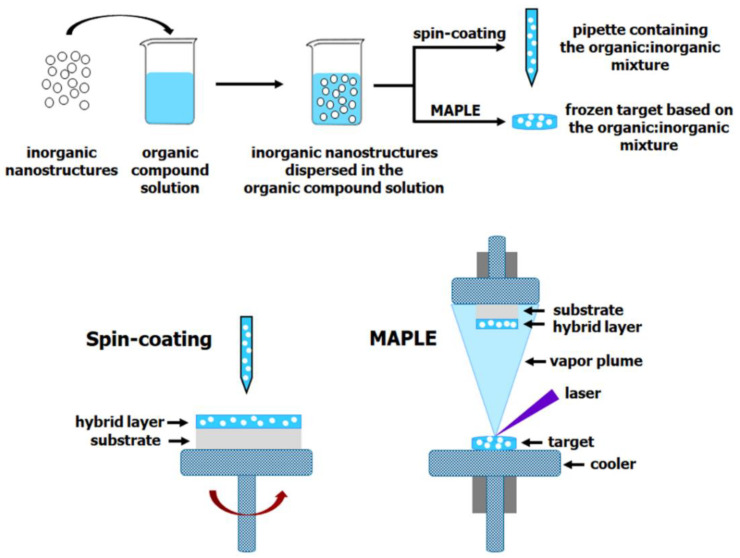
Schematic illustration of the steps involved in the deposition of organic:inorganic hybrid composite layers by spin-coating and MAPLE techniques.

The deposition of hybrid composite layers by both spin-coating and MAPLE techniques used similar steps with those described in the deposition of the organic layers with the difference that the inorganic nanostructures, synthesized previously by various chemical routes, are added in the organic solution. Thus, in the case of spin-coating, the hybrid film is deposited directly from the organic:inorganic mixed solution, whereas, in the case of MAPLE, the organic:inorganic mixed solution is frozen for preparing the solid target which will be further used for the hybrid film deposition. In Figure 4 is presented a schematic representation of the steps regarding the deposition of the thin films based on hybrid composites by both spin-coating and MAPLE techniques.

## 4. Hybrid Nanocomposite Films Deposited by Spin-Coating for PV Cells

Spin-coating is still the most widely used approach which allows a quick and easy deposition of the layers involved in the fabrication of the OPV or HPV devices. The various hybrid composite layers based on inorganic nanostructures, such as metal oxides, chalcogenides, and silicon, which were deposited by spin-coating in order to be integrated as active layers or as additional buffer layers in the PV devices will be further presented in a chronological order of the publication date to help readers through their reading.

### 4.1. Metal Oxides Based Nanocomposites

#### 4.1.1. ZnO

In the field of the hybrid PV cells based on ZnO nanostructures, the most common active layers involved P3HT or P3HT:PCBM blends.

Oosterhout (2011) prepared and investigated PV cells based on hybrid films containing ZnO nanostructures and P3HT or its ester-functionalized side chain poly(3-hexylthiophene-2,5-diyl) derivative (P3HT-E) [100]. Although, the morphological investigations showed that the ZnO nanostructures are much better dispersed in P3HT-E than in P3HT, the presence of this derivative compound featured by a low degree of crystallinity seems to reduce the connectivity between the semiconductor nanoparticles that finally results in a decrease of the hole mobility compared to the blend containing P3HT as donor component, the best cell performance (1.7%) being recorded for the structure based on this conjugated polymer and ZnO.

Bhat (2011) involved a composite based on ZnO nanoparticles and P3HT as the active layer in the fabrication of stable in air fullerene-free hybrid PV cell structure [102]. A cell efficiency of 1% is achieved for a structure using, besides the active layer, a ZnO film as an electron selective layer and a PEDOT:PSS supplementary layer.

Rakshit (2012) used ZnO nanorods and CdS-decorated ZnO nanorods in order to obtain hybrid films with P3HT involving different casting solvents (chlorobenzene, toluene, chloroform) [175]. The study revealed that (i) the increase of the ZnO concentration to a certain amount in the prepared films leads to an increase of the cell efficiency (0.12%); (ii) the casting solvent type has a strong impact on the efficiency of the cell; and (iii) the functionalization of the ZnO nanorods surface with CdS nanoparticles results in an increase of the device efficiency (0.38%) due to the formation of a better cascade band structure which favors the charge transfer at the electrodes.

Hu (2013) integrated a composite film based on poly(ethylene glycol) (PEG) and ZnO nanoparticles (working as CBL) into a structure having P3HT:PCBM active layer, the device being fabricated on a flexible substrate (poly(ethylene terephthalate)) [176]. The influence of the PEG molecular weight (Mw) on the electrical performances of the prepared PV structures was analyzed, a short polymer backbone (Mw = 400) being inefficient in the passivation of the ZnO surface traps whereas a long polymer backbone (Mw = 20,000) generating a charge transport barrier due to the insulating PEG behavior. Thus, the use in the hybrid layer of the ZnO nanoparticles coated with PEG featured by an optimum of lone electron pairs of oxygen in its backbone (Mw = 6000) leads to an increase of the cell efficiency (3.3%) in comparison with that recorded for the cell containing only ZnO nanoparticles (2.3%). The improvement is attributed to the passivation of the ZnO surface traps lowering the probability of the charge recombination and to the improvement of the alignment of the energy level at the interface between ZnO and PC61BM.

Zhong (2014) prepared an HPV cell structure with Z907-modified ZnO nanorods and P3HT that exhibits 0.21% efficiency [177]. In this case, the ruthenium dye molecule Z907 grafted onto the ZnO surface improves the compatibility between ZnO nanostructures and P3HT, does not substantially modify the ZnO crystalline structure, expands the visible light absorption range of the composite film, and enhances the charge transfer efficiency at the organic:inorganic interface.

Anjum (2015) added ZnO nanoparticles to P3HT:PCBM blend in order to obtain mixed layers, various cell configurations (regular or inverted) with PEDOT:PSS or TiO_2_ acting as buffer layers being evaluated [178]. In both structure types, the presence of the ZnO nanoparticles has as a consequence an improvement in the cell efficiency, 0.77% for the structure with PEDOT:PSS and 0.63% for that with TiO_2_, due to the increased absorption.

**Figure 5:**
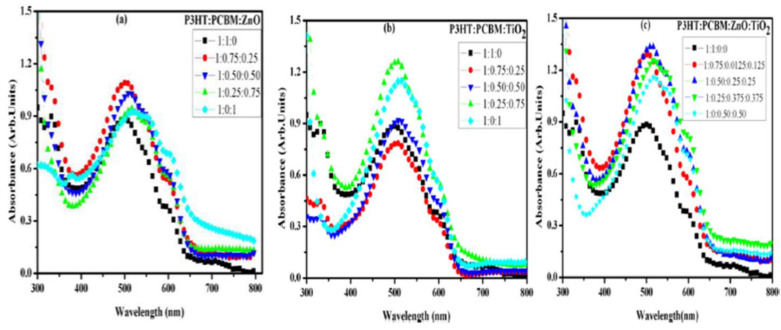
Optical absorbance spectra of hybrid films based on (**a**) P3HT:PCBM:ZnO, (**b**) P3HT:PCBM:TiO_2_, and (**c**) P3HT:PCBM:ZnO:TiO_2_ blends with various weight ratios: 1:1:0, 1:0.75:0.25, 1:0.50.0.50, 1:0.25:0.75, 1:0:1 for blends based on ZnO nanoparticles (**a**) or TiO_2_ nanoparticles (**b**) and 1:1:0:0, 1:0.75:0.125:0.125, 1:0.50:0.25:0.25, 1:0.25:0.375:0.375, 1:0:0.50:0.50 for blends based on a ZnO and TiO_2_ nanoparticles mixture (**c**). Reprinted with permission from [94]. Copyright 2015 AIP Publishing.

Ikram (2015) investigated the way in which the properties of hybrid films based on P3HT:PCBM:ZnO nanoparticles are influenced by the component amounts, keeping constant the P3HT amount and varying the PCBM and ZnO amounts [94]. The following effects were put in evidence: (i) The addition of an optimum amount of ZnO nanoparticles in the active layer improves the absorption (Figure 5a) and the charge carrier mobility; (ii) the efficiency of the inverted cell structure increases from 2.69% to 2.96% when a small amount of ZnO is added in the blend (1:0.75:0.25); and (iii) the efficiency value decreases when the ZnO amount is increased above the optimum concentration, the effect being correlated to the nanoparticles agglomeration tendency and to the increased roughness of the active layer. It must be mentioned that the hybrid films based on P3HT:PCBM:TiO_2_ (Figure 5b) and P3HT:PCBM:ZnO:TiO_2_ (Figure 5c) were also investigated in this study, the results being analyzed in the section focused on TiO_2_.

Muller (2016) reported on the incorporation of ZnO nanoparticles in P3HT:PCBM blend in an inverted cell structure having TiO_2_ and MoO_3_ as additional layers [179]. The results showed that the thermal stability of P3HT was improved after incorporation of ZnO in the polymer matrix. For the cell based on P3HT:PCBM:ZnO composite, an increase in the efficiency from 1% to 1.7% was obtained at specific ZnO nanoparticle amounts and a certain ratio between the composite components (1:1:1). An excessive ZnO amount decreases the cell efficiency due to a large phase separation between the three constituents, this effect being induced by the nanoparticles agglomeration process, formation of a rougher surface and increase of the layer thickness.

Li (2020) studied a composite based on ZnO and 2,3,5,6-tetrafluoro-7,7,8,8-tetracyanoquinodimethane (F4-TCNQ) that was integrated as a cathode buffer layer (CBL) in two device architectures involving fullerene (PC71BM) or non-fullerene (ITIC) compounds as acceptors [96]. An increase in the cell efficiency from 7.17% to 8.14% was obtained for the cell prepared with 1% F4-TCNQ compared to that prepared only with ZnO. Additionally, for the same F4-TCNQ content, an improvement of the cell efficiency from 6.13% to 6.96% was remarked for the structure using ITIC instead of PC71BM. The improvement of the electrical properties of the assessed structure was attributed to the optimized energy levels in the CBL layer due to the F4-TCNQ presence and to the trap-filling strategy: F4-TCNQ filled the ZnO defects reducing the trap-assisted recombination in the CBL layer and allowing an efficient charge transport.

#### 4.1.2. CuO

Hybrid PV cells based on CuO nanostructures usually employed P3HT:PC70BM or P3HT:PCBM blends in the active layers.

Wanninayake (2015) reported on the incorporation of the CuO nanoparticles within P3HT:PC70BM to be used as an active layer in PV cells [180]. The investigated solar cell structures containing various amounts of CuO nanoparticles showed a higher efficiency than that of the cell prepared only with organic materials. A cell efficiency of 2.96% is recorded for the cell containing an optimum CuO nanoparticles content in comparison to 2.1% obtained for the reference cell (without metal oxide nanoparticles). The result was explained taking into account that the addition of CuO leads to a better absorption of the active layer, an increase of the exciton diffusion coefficient due to high hole and electron mobility, an increase of the exciton dissociation, and a better pathway in the composite blend for the charge transport process.

Ikram (2015) prepared and evaluated cell structures with active layers based on ternary blends containing P3HT:CuO:PCBM [181]. Similar with the other studies, the optimum CuO amount required for improving the cell efficiency was found by varying the ratio between P3HT and CuO nanoparticles within the active layer. Hence, a cell efficiency of 3.7% was achieved for the cell based on organic:inorganic blend, the parameter value being higher than that obtained for the reference cell (P3HT:PCBM). Additionally, the increase of the CuO amount in the active layer results in a redshift and an enhancement of the absorption, and also in an improvement of the P3HT crystallinity.

Ikram (2015) also added ZnO nanoparticles as an additional acceptor material within the P3HT:CuO:PCBM blend (0.5:0.5:0.5:0.5) [182]. Thus, the cell efficiency reached 4.09%, being attributed to the enhancement of the absorption and to the increase of the surface roughness of the active layer.

Wanninayake (2016) added various amounts of ZnO nanoparticles in an electron transport buffer layer deposited on top of the active layer containing a certain amount (0.6 mg) of CuO nanoparticles having 35–50 nm in size and a P3HT:PCBM blend (1:1) [183]. The solar cells prepared only with organic materials and ZnO as a buffer layer achieved 2.54% efficiency while all the other cells fabricated with CuO nanoparticles inside the active film reached higher efficiency, the best value (3.95%) being obtained for the cells containing an optimum amount (20 mg) of ZnO nanoparticles. Again, the inorganic nanoparticles enhance the device performance due to the increase of the optical absorption and improvement of the exciton dissociation.

**Figure 6:**
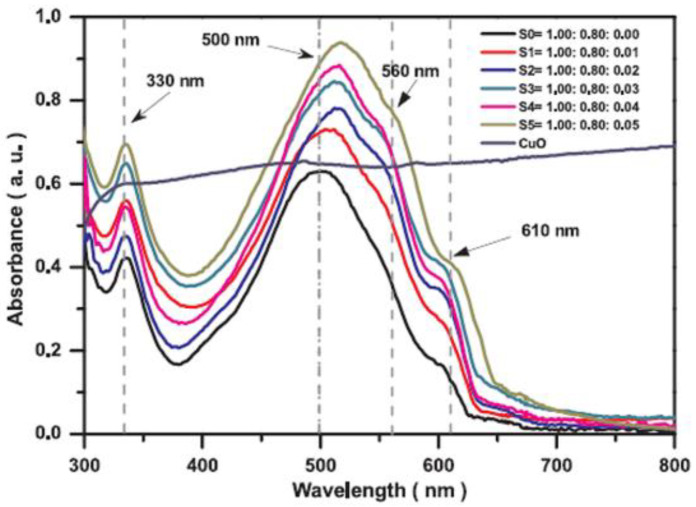
UV–VIS absorption spectra of hybrid films based on P3HT:PCBM:CuO blends with various weight ratios, the amount of CuO nanoparticles increasing from bottom to top: 1:0.8:0.0 (S0), 1:0.8:0.01 (S1), 1:0.8:0.02 (S2), 1:0.8:0.03 (S3), 1:0.8:0.04 (S), and 1:0.8:0.05 (S5). Reprinted with permission from [184]. Copyright 2019 Elsevier.

Salim (2019) reported on the incorporation of CuO nanoparticles in P3HT:PCBM blends [184]. Thus, the addition of a certain CuO amount increases the efficiency from 3% for the cell without metal oxide nanoparticles at 4.1% for the cell based on P3HT:PCBM:CuO (1.00:0.80:0.03), the increase of the electrical parameters being once more correlated with the improvement of factors such as optical absorption (Figure 6), crystallinity and roughness of the active layer.

Siddiqui (2020) developed PV cells by incorporating CuO nanoparticles in different ratios (from 1wt% to 10 wt%) within P3HT:PC70BM blends [185]. If the cells without CuO exhibit 2.85% efficiency, the addition of an optimum CuO amount (5 wt%) enhance the cell efficiency to 3.82%. The inclusion of the inorganic nanoparticles into the organic blend results in an absorption shift to the visible region which, in turn, is responsible for a better absorption of light and an efficient generation of the photo-excited charges.

#### 4.1.3. TiO_2_

In the case of the hybrid PV cells based on TiO_2_ nanostructures, the active layers can involve a P3HT:PCBM blend, polycarbazole-based conjugated polymer, or other polymers like PPV, doped polyaniline (PANI), etc.

Dong (2012) developed PV cells using anatase TiO_2_ nanocrystals and PPV analyzing the influence of the weight ratio between the two components on the device performance [186]. The best efficiency (0.11%) was recorded for the cell with the active layer based on a PPV:TiO_2_ blend ratio of 1:3, an optimized morphology with bicontinuous interpenetrating network of PPV and TiO_2_ being obtained for this ratio value.

Thao (2015) prepared PV cell structures based on P3HT:PCBM:TiO_2_ [187]. The cells containing the composite layer achieved a higher efficiency (1.73%) in comparison with that obtained for the cells prepared only with the organic materials (1.45%). Moreover, it is shown that an annealing treatment can enhance the cell efficiency, reaching at 2.1% for the HPV cell after annealing at 70 °C and at 1.6% for the OPV cell after annealing at 60 °C. The results confirm that the PV cells based on composites are featured by a better thermal stability than PV cells based only on organic compounds.

Ikram (2015) incorporated TiO_2_ nanoparticles within the P3HT:PCBM blends [94]. A cell efficiency of 3.10% was recorded for those containing the composite in the active layer while a cell efficiency of 2.69% was obtained for those using only P3HT:PCBM in the active film. Similar to the incorporation of other metal oxide (ZnO, CuO) nanoparticles into organic compounds of the active layers developed for the PV applications, the addition of an optimum TiO_2_ nanoparticles amount increases the P3HT crystallinity, the absorption in the visible region (Figure 5b) and the charge carrier mobility leading to an enhancement of the cell efficiency. Instead, the addition of a higher amount of TiO_2_ nanoparticles results in a decrease of the cell efficiency due to the increase of the surface roughness and to the aggregation of the metal oxide nanoparticles. By adding TiO_2_ and ZnO nanoparticles in an equal amount (Figure 5c) within the P3HT:PCBM blends (1:0.75:0.125:0.125), the cell efficiency reached at 3.03%, an intermediary value relative to those recorded for the structures having a single type of inorganic nanoparticles, 2.96% for ZnO and 3.10% for TiO_2_. It has to be noted that the metal oxide nanoparticles are completely agglomerated in the absence of PCBM, the outcome suggesting that the fullerene derivative compound can hindered the aggregation tendency of the inorganic nanostructures.

Geethalakshmi (2016) integrated films based on TiO_2_ nanoparticles and camphorsulfonic acid doped PANI in the solar cell’s structures [188]. Analyzing the effect of TiO_2_ addition in different weight ratio on the properties of the prepared structures, the best cell efficiency (0.21%) was determined for the cell having a lower quantity of TiO_2_ (1:8:2). The study emphasized that such structures based on a single layer of PANI-TiO_2_ BHJ can replace the combination of liquid electrolyte (usually employing sealing issues), dye sensitizer, PANI layer, and TiO_2_ layer.

Yin (2015) developed nanocomposite films based on PEG and titanium oxide for acting as CBL in polymeric inverted solar structures, the goal of the study being the improvement of the device performance by enhancing the charge collection and reducing the energy barriers formed at the organic:inorganic interface [91]. The insertion of these hybrid films having ~10 nm in thickness in the solar cell structures based on PDTTBT-3, PTB7, PTB7-Th, and PC71BM has as consequence a significant improvement of the electrical parameters of the fabricated devices. Thus, the efficiency was 2.80% for the reference cell (without buffer layer) while the CBL presence increases the cell efficiency to 5.14%, 8.72%, and 9.05% for cells based on PDTTBT-3, PTB7, and PTB7-Th, respectively.

#### 4.1.4. Cr_2_O_3_

Amber Yousaf (2018) studied the effect of the addition of Cr_2_O_3_ nanoparticles (10–30 nm) in the active layers based on P3HT:PCBM or PTB7:PCBM [189]. Hence, the presence of the inorganic nanoparticles leads to the enhancement of the light harvesting and tuned energy levels, for the devices based on P3HT:PCBM, the cell efficiency being increased from 2.51% (reference cell) to 3.67% (the cell having 3% Cr_2_O_3_ content), while for those prepared with PTB7:PCBM, an improvement from 5.61% (reference cell) to 6.72% (the cell having 2% Cr_2_O_3_ content) being achieved.

### 4.2. Chalcogenides Based Nanocomposites

#### 4.2.1. CdSe

Undoubtedly, CdSe is the chalcogenide compound most frequently used for developing organic:inorganic hybrid cells, its colloidal nanostructures being usually embedded in a polymer matrix (most often P3HT, but the potential of other polymers, such as PCDTBT or PCPDTBT, was also evaluated). Additionally, the performance of the hybrid solar cells based on CdSe nanocrystals can be improved by replacing the insulating coating ligands originating from the semiconductor colloid synthesis (which are not adequate for PV applications) with smaller molecules, such as pyridines [190], amines [191,192], thiols [193], etc., in order to enhance the charge transfer. Therefore, the PV devices based on CdSe nanocrystals can reach high efficiency values compared with other structures based on inorganic nanostructures [155].

Brandenburg (2011) studied the effect of the CdSe QDs particle size (2–3 nm, 3–4 nm and 10 nm) on the properties of the PV structures involving the same conjugated polymer (P3HT) as donor component [194]. Hence, the QDs size have a major impact on the V_OC_, an increase by nearly 50% of this parameter being obtained when smaller CdSe ODs were used. Taking into account that the largest nanoparticles have the strongest aggregation tendency in colloidal solution, the best cell efficiency (0.45%) was reached for the structure employing CdSe QDs with 3.9 nm in diameter.

Lee (2011) used tetrapod shaped CdSe nanoparticles (long-armed with 10 nm length and 4 nm width or short-armed with 5 nm length and 4 nm width) to prepare hybrid films involving P3HT as the polymer matrix [99]. The study reveals that a better spatial connectivity is obtained between the two components of the blends when longer armed tetrapods are used, this effect enhances the charge transport in the PV cell. Thus, an improvement in the cell efficiency is obtained for the structure using 10 nm long-armed tetrapods (1.12%) in comparison with the structure using 5 nm short-armed tetrapods (0.80%).

Qian (2011) also investigated the influence of the CdSe nanocrystals size (5 nm and 6.5 nm) added into the blend layer and the addition of a ZnO nanoparticles (3–4 nm in size) buffer layer on the properties of the PV structures fabricated with P3HT as donor material [195]. The best cell efficiency (2.2%) was achieved for the structure containing CdSe nanocrystals with 6.5 nm in diameter in the active layers and the ZnO supplementary layer. The presence of the ZnO nanoparticle layer reduces the leakage of the photogenerated charge carriers towards cathode, enhances the harvesting of the photogenerated excitons in the active layer and improves the air stability of the PV cell.

Radychev (2011) developed PV cells based on alkylamines-capped CdSe QDs (3.4 nm in size) and P3HT [196]. Additionally, in this case, a ligand exchange process was applied, the native oleic acid ligands from the CdSe QDs surface being exchanged with alkylamines ligands, more suitable for the applications of the CdSe QDs in the solar cells area. The binding of the amine molecules is responsible for a better surface passivation improving in this way the charge transport in the hybrid blend. The influence of the thickness of the active layers and the thermal annealing temperature on the solar cell performances was studied, the best cell efficiency (2.0%) being achieved for the structure having a 75 nm active layer thickness treated at 180 °C.

**Figure 7:**
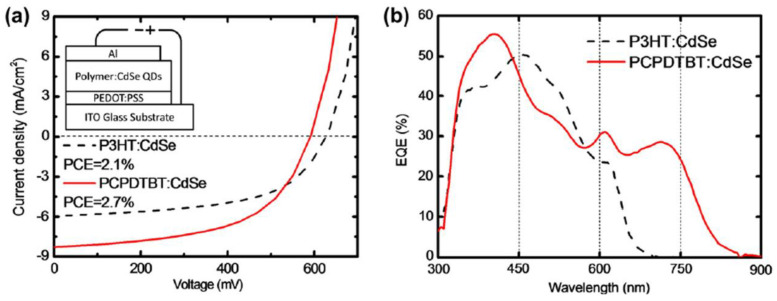
(**a**) J-V characteristics under illumination of the PV cells fabricated with hybrid films based on P3HT:CdSe or PCPDTBT:CdSe blends. Inset: schematic representation of the PV architecture. (**b**) EQE spectra of the corresponding solar cells. Reprinted with permission from [197]. Copyright 2011 Elsevier.

Zhou (2011) fabricated hybrid PV cells based on CdSe QDs (4.7 nm in size) and P3HT or PCPDTBT as polymer matrix [197]. A surface modification process regarding the replacement of the insulating ligands from the CdSe QDs surface was again made in order to improve the cell performance. Thus, a hexanoic acid treatment was applied to the trioctylphosphine/oleic acid-capped CdSe QDs before their incorporation in the conjugated polymer. Moreover, using PCPDTBT, a low band gap polymer instead of P3HT, a higher efficiency (2.7%) was obtained, this behavior being mainly attributed to the increase of the J_SC_ related to a better match of the blend absorption with the solar emission spectrum (Figure 7).

Greaney (2012) investigated the electrical performances of the PV cells based on CdSe nanocrystals (4.5 nm in size) and P3HT [198]. The study evidenced that an increase in the device performance (1.9% cell efficiency) is attained by exchanging the large and insulating stearate alkylphosphonates ligand, from the surface of the CdSe nanocrystals, with the small, strongly binding and electron donating tert-butylthiol ligand, their presence resulting in a better charge transfer between the donor and acceptor components and charge collection from the semiconductor nanocrystals phase.

Lek (2012) developed hybrid PV cells with pyridine-capped CdSe nanorods (52 nm length and 9 nm width) and P3HT or PCDTBT as polymer matrix [199]. After the optimization of the amount of the pyridine-capped CdSe nanorods added in the conjugated polymer and of the active layer thickness, the cell efficiency was 2.17% for the structure using P3HT and 2% for that involving PCDTBT, the last one having a deeper HOMO level relative to P3HT. Additionally, the PV cell based on PCDTBT is characterized by a 35% increase in V_OC_ compared to that using P3HT.

Jeltsch (2012) fabricated hybrid photovoltaic cells using PCPDTBT and two types of CdSe nanostructures, QDs (4.7 nm in size) and nanorods (20–30 nm in length) evaluating the influence of the polymer:nanocrystal loading ratio and the annealing temperature on the solar cells parameters [200]. For the devices based only on CdSe QDs, the best cell efficiency (2.8%) is reached for a hybrid layer with 90 nm thick active layer treated at 210 °C and having 10:90 ratio between PCPDTBT and CdSe QDs, the interconnectivity between the semiconducting QDs being enhanced by the applied annealing temperature. The cell efficiency increased to 3.6% for the devices based on CdSe QDs and a CdSe nanorod mixture, the improvement being related to the better connection between the small spherical quantum dots and the mostly in plane aligned elongated nanorods which leads to the formation of a well-interconnected *n*-type network that favors the electron transport and reduces the recombination processes within the *p*-type polymer matrix.

Kim (2012) prepared composite layers based on CdSe QDs and a poly(3-hexyl thiophene)-block-poly(N,N-dimethylamino-2-ethylmethacrylate) (P3HT-b-PDMAEMA) block copolymers [201]. The study has shown that an interpenetrating networks is formed between the two components, a cell efficiency of 0.1% being recorded.

Kwon (2013) used selenourea (SeU) in the ligand exchange process for improving the performance of the devices containing the composite films based on P3HT and CdSe nanorods [202]. Hence, selenourea is easily decomposed into selenide during the annealing at 215 °C of the composite film. After the addition of an appropriate selenourea amount, the interconnectivity between the CdSe nanorods in the polymer matrix is preserved and no dramatically aggregation effect is observed in the hybrid layer. Therefore, the efficient charge hopping through the formed network results in an enhancement of the charge transport. A cell efficiency of 2.63% is recorded for the PV device based on P3HT:CdSe with selenourea, which means an increase by 54% relative to that obtained for the device without selenourea.

Zhou (2013) applied a chemical treatment to the hybrid films based on CdSe nanorods (32 nm in length and 4.4 nm in width) and P3HT or PCDTBT as polymer matrix for increasing the device performance [203]. Thus, during the immersion of the hybrid layer in an ethanedithiol-containing acetonitrile, the surface defects on CdSe nanorods is passivated due to the removal of the charged surface ligands. The best cell efficiency (4.7%) of the structure involving PCDTBT and CdSe nanorods is related to the low band gap of PCDTBT and better polymer:inorganic nanorods interface leading to an increase in the charge generation and transport and to a decrease in the exciton and charge carrier recombination.

Fu (2014) attached benzenedithiol ligands on the surface of CdSe nanocrystals (6 nm in size) for improving the performance of the hybrid PV cells based on PCPDTBT and CdSe QDs, with a buffer layer of poly[(9,9-bis(3′-(N,N-dimethylamino)propyl)-2,7-fluorene)-alt-2,7-(9,9-dioctylfluorene)] (PFN) working as CBL [204]. Hence, by a ligand exchange process, the benzenedithiol ligands are attached onto the surface of the CdSe QDs in a “face-on” geometry in order to minimize the nanocrystal-nanocrystal or polymer-nanocrystal distance. Thus, an enhanced electronic coupling between the nanocrystals and the benzene ring oriented “face-on” at the interface is achieved, this facilitating the exciton dissociation at the polymer:semiconductor QD interface and the charge transport. The cell efficiency of 4.18% recorded for the structure based on PCPDTBT:CdSe QDs when benzenedithiol is used for ligand exchange is attributed to the ligand orientation effects.

Benchaabane (2016) prepared hybrid films based on poly(3-octylthiophene) (P3OT) and oleic acid-capped CdSe nanoparticles (8 nm in size), investigating the influence of the CdSe nanoparticles amount on the PV cell performance [205]. Thus, the cell efficiency was improved from 0.025% for the cell with pure P3OT to 0.51% for the cell containing 80% CdSe nanoparticles. The incorporation of the semiconductor nanoparticles leads to a photoluminescence quenching and exciton dissociation enhancing, the charge separation taking place at the P3OT:CdSe nanoparticles interface.

Dayneko (2016) analyzed the influence of the CdSe nanoparticles size (5 nm or 10 nm) on the properties of hybrid layers based on PCDTBT:CdSe or PTB7:CdSe [206]. The best photocurrent density values, 0.25 mA/cm^2^ and 0.35 mA/cm^2^ were recorded for PCDTBT:CdSe and PTB7:CdSe, respectively, both structures containing CdSe QDs with 10 nm in size. Thus, by tuning the size of the CdSe nanoparticles can be optimized the relative position of the energy levels in the materials involved in the development of the PV cell leading to the improvement of the device performance.

Xu (2017) developed hybrid active layers based on P3HT nanowires (15 nm in diameter) and CdSe nanotetrapods (20–25 nm arm length and 4–5 nm arm diameter) [207]. A 1.7% cell efficiency was reached for the structure involving P3HT nanowires as the electron donor and CdSe nanotetrapods as the electron acceptor, meaning an enhancement of ~42% in respect to the efficiency recorded for the traditional cell using dissolved P3HT molecules. The improvement was associated to the broad light absorption linked to a well crystallized P3HT and to the more efficient charge generation and transport through the bicontinuous charge channels in this hybrid based on the two nano-building blocks (nanowires and nanotetrapods).

Madsuha (2019) used hexadecylamine-capped CdSe QDs (6 nm in size) and PCPDTBT for preparing PV cells, for improving the device performance, a hexanoic acid washing treatment being applied to the CdSe QDs before their incorporation in the polymer matrix [208]. Thus, using an optimum (22 min) time washing with hexanoic acid, before the CdSe QDs addition within the photoactive layer of the solar cells, the insulating ligand was removed from their surface, the electron transport in the hybrid film was improved and a cell efficiency of 2.81% was recorded for the fabricated device.

Nabil (2020) used tetra butyl ammonium iodide-capped CdSe QDs (4.25 nm in size) and P3HT:PCBM for developing solar cells [209]. The cell efficiency of 1.99% recorded in the case of the reference cell increased to 2.5% in the HPV cell due to the band matching between the inorganic QDs and organic materials and the enhanced sunlight absorption in the visible range.

Kumar (Kumar 2020) developed PV cells by adding CdSe QDs (4.48 nm) in the active layers based on PCDTBT:PC61BM blends and using MoO_3_ and ZnO QDs (2.87 nm) layers as transparent hole transport layer and electron transport layer, respectively [210]. An important increasing in the cell efficiency value from 3.62% for the reference cell based only on organic layers to 5.02% for the ternary cell was achieved. The result was related to the presence of CdSe QDs, the synergetic effect between CdSe QDs and PC61BM resulting in an increase of the light absorption and the charge transportation.

Yang (2021) involved CdSe QDs (4.2 nm in size), in different ratios, and PTB7-Th:PC71BM blend for obtaining hybrid films for PV cells [211]. The presence of the CdSe QDs increased the absorbance and the fluorescence external quantum efficiency, an improvement in the cell efficiency from 8.58% recorded for reference cell to 9.57% being achieved for the cell containing CdSe QDs in an optimum amount (5% loading). Forster resonance energy transfer from CdSe QDs to the donor also plays an important role in the device performance.

#### 4.2.2. CdTe

Indisputably, CdTe has become an attractive choice for the development of the PV cells based on its colloidal nanostructures because these are usually synthesized by aqueous solution-based routes (2-mercaptoethylamine being the commonly coating ligand used for stabilizing the colloids) [169]. Furthermore, the conjugated polymers (PPV, MEH-PPV, poly(1,4-naphthalenevinylene) (PNV), P3HT) often used as the polymer matrix for the CdTe nanocrystals in the PV cells are prepared from water-soluble precursors. Consequently, an aqueous-solution-processed hybrid solar cells can be fabricated by more environmentally friendly paths in comparison with those frequently used for the devices obtained from organic soluble materials.

Fan (2011) prepared blends based on CdTe nanocrystals (3 nm in size) and PNV for integrating them in hybrid solar cells [212]. Hence, using a proper ratio between the PNV and CdTe nanocrystals (1:18), a continuously interpenetrating network was formed between the two components resulting in a cell efficiency of 0.86%.

Chen (2013) used CdTe nanocrystals and PPV for developing inverted hybrid solar cells with different architectures [213]. Thus, designing various cell architecture, the cell efficiency was enhanced as follows: 3.64% for the cell containing BHJ structure; 3.75% for the cell using a planar heterojunction in which the CdTe film is introduced between the TiO_2_ layer and the PPV layer; 3.82% for the cell having a *n*-*i* structure in which the PPV layer is replaced by a hybrid layer based on PPV and CdTe nanocrystals; 4.76% for the cell featured by the same *n*-*i* structure in which a very thin CdCl_2_ film is inserted between the CdTe film and the hybrid layer for improving the hydrophilicity of the annealed CdTe film.

Yao (2015) blended CdTe nanocrystals (3.32 nm in size) with P3HT dots (2.09 nm in size) in order to obtain a composite layer for integrated it as active film in the PV cells which involved also a supplementary CdTe nanocrystals layer [214]. The best cell efficiency (4.32%) was recorded for the structure based on an active layer with 1:24 donor-acceptor ratio, thermally treated at 265 °C. The annealing temperature results in an increase of CdTe nanocrystals size, but this effect was limited by the addition of P3HT dots, the presence of the polymer decreasing the annealing time from 10 h to 1 h. The parameters used in the thermal annealing have the main impact on the device performance taking into account that it facilitates the formation of a smoother interpenetrating network in the active layer which further improves the charge separation and the charge transport in the composite layer.

Liu (2016) prepared and investigated composite layers based on CdTe nanocrystals and MEH-PPV [133]. The study shown that the miscibility between the polymer matrix (MEH-PPV) and the inorganic colloids from aqueous media (CdTe nanocrystals) can be improved by using a water-soluble precursor. After the annealing process, between the inorganic nanocrystals and the polymer matrix is formed an interpenetrating network structure. Hence, a 4.2% cell efficiency (comparable with those recorded for oil-processed) was recorded for the structure based on an active layer with organic:inorganic 1:12 ratio, thermally treated at 315 °C. Again, the annealing temperature has a major impact on the charge transfer and transport taking into account that it plays an important role in the stability of the conjugated polymer and of the size of the inorganic nanocrystals: lower annealing temperatures do not favor the appearance of the interpenetrating network (the inorganic nanocrystals remain dispersed in the polymer matrix), while higher annealing temperatures decompose the polymer and increase the size of nanocrystals which results in a phase separation that affects the device performance.

Jin (2017) used CdTe nanocrystals (18 nm in size), TiO_2_ nanocrystals (15 nm in size) and PPV in the development of PV cells with double-side BHJ [215]. In such a structure, one BHJ combines the active material with electron transport material and the other combines the active material with hole transport material. Thus, the CdTe layer is simultaneously sandwiched by the TiO_2_:CdTe and CdTe:PPV BHJ films, a cell efficiency of 6.01% being recorded due to the enhancement of the carrier extraction and transport length.

Chen (2019) employed CdTe nanocrystals (18 nm in size), ZnO nanocrystals (35 nm in size) and PPV in the development of PV cells with the same double-side BHJ architecture [216]. This time, the CdTe layer is simultaneously sandwiched between the ZnO:CdTe BHJ (increase the electron extraction ability) and the CdTe:PPV BHJ (increase the hole extraction ability). A supplementary ZnO layer showing better transmittance and higher electrical conductivity is also used in this PV cell contributing to charge carrier generation and transport. For comparison reason, a PV cell using the double-side BHJ but involving TiO_2_ instead of ZnO was fabricated. A 5.87% cell efficiency was recorded for the PV cell using TiO_2_. The best cell efficiency, 6.51%, was obtained for the device containing ZnO, this BHJ configuration being responsible for the enhancement in the light absorption and in the carrier extraction ability as well as for the increase of the depletion region width and of the carrier lifetime.

#### 4.2.3. CdS

The potential applications of CdS in the PV area are due to its absorption in the visible region of the spectrum.

Inpor (2011) prepared and investigated HPV cell structures based on MEH-PPV:CdS nanorods blends and a nanostructured TiO_2_ layer [103]. A 0.23% cell efficiency was recorded for the structure involving organic:inorganic blend relative to 0.1% determined for the reference structure having only MEH-PPV as active layer. The enhancement in the cell efficiency was associated with a more stable depletion region at the interface between MEH-PPV and TiO_2_ layer due to the presence of CdS nanorods in an optimum amount, a higher content leading to an agglomeration process of CdS nanoparticles that further reduces the device performance.

**Figure 8:**
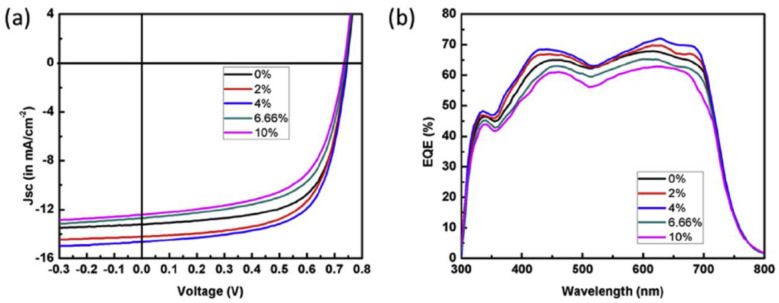
(**a**) J–V characteristics and (**b**) EQE spectra of PV cells fabricated with hybrid layers based on PTB7:PCBM:CdS blends with different weight ratio of CdS nanoparticles. Reprinted with permission from [121]. Copyright 2016 Elsevier.

Sharma (2016) developed PV cells, in an inverted geometry, based on ternary hybrid blends containing CdS elongated nanoparticles (6–10 nm in diameter and 10–20 nm in length), PTB7, and PCBM, evaluating the influence of the CdS amount on the device performance [121]. Again, the best cell efficiency (7.01%) was achieved for the cell having an optimum inorganic nanoparticles concentration (4%), a higher CdS concentration inducing a decrease in the device performance (Figure 8).

Aruna-Devi (2021) investigated the effect induced by the morphology of the CdS nanostructures (irregular shaped agglomerated nanoparticles, spherical nanoparticles with sizes varying from 5.5 to 13 nm and nanorods with 12 nm length and 4 nm width) on the properties of PV cells based on a P3HT:PCBM blend and these inorganic nanostructures [217]. Thus, the addition of CdS nanostructures with various shapes increases the efficiency from 1.2% for the cell without inorganic compound at 1.65% for the cell containing hybrid layer with CdS spherical nanoparticles and reaches at 2.42% for the cells containing CdS nanorods in the hybrid layers. The improvement in the cell efficiency was linked to the presence of the inorganic nanostructures: Spherical nanoparticles can lead to an increase of the interface area between the donor and acceptor that facilitates the charge separation while the nanorods, due to their aspect ratio, can give a continuous pathway for the charge transport process.

#### 4.2.4. PbSe

Tan (2011) obtained composite layers based on PbSe QDs (~6 nm in size) and P3HT, these being further involved in the fabrication of infrared solar cells structures [218]. The study has shown that the presence in the PV structure of a supplementary P3HT layer allows a more efficient carrier collection without a significant decrease of the light absorption in the device active region influencing the device performance. A cell efficiency of 0.26% was recorded for this structure instead of 0.1% measured for the reference cell.

#### 4.2.5. PbS

Lu (2016) prepared iodine-passivated PbS nanocrystals (3.2 nm in size), these inorganic nanostructures and Si-PCPDTBT (a donor polymer) being further used in the development of the infrared solar cells structures [125]. Thus, a simple colloidal ligand exchange process for PbS nanocrystals based on iodine ligands from PbI_2_ and NH_4_I allows them to be dispersed in solvents compatible with polymer dissolution. The best cell efficiency (4.8%) reported for the structure using the composite film based on PbI_2_-exchanged PbS as acceptor was related to a more efficient charge transfer process in comparison with that involved in the structure using NH_4_I-exhanged PbS.

Thomas (2020) fabricated an inverted bulk-heterojunction PV cell based on PbS nanocrystals and P3HT blends, with a ZnO nanocrystals layer (ETL) and an additional buffer layer containing CdSe QDs between the ZnO layer and the active layer [219]. In the absence of the CdSe layer, the cell efficiency is 1.7% while the insertion of this layer increased the cell efficiency to 2.4%. Thus, the CdSe layer provides as an intermediate energy band at the interface formed between the active layer and the ZnO layer, reducing the recombination process at the interface by passivating the ZnO surface states and favoring the electron transfer.

Baek (2019) used 1-ethyl-3-methylimidazolium iodide-capped PbS QDs, a copolymer of benzo[1,2-b:4,5-b’]dithiophene and thieno[3,4-b]thiophene (PBDTTT-E-T) and a small molecule 2,2′-((2Z,2′Z)-((5,5′-bis(4,4,9,9-tetrakis)-4,9-dihydros-indaceno[1,2-b:5,6-b’]dithiophene-2,7-diyl)bis(4-(2-ethylhexyl) oxy)thiophene-diyl)bis (methanylylene))bis-(3-oxo-2,3-dihydro-1H-indene-2,1-diylidenene)dimalononitrile (IEICO) for developing PV cells [220]. The devices prepared with these composite layers achieved 13.1% cell efficiency due to the presence of IEICO. This small molecule has complementary absorption to inorganic QDs and forms an exciton cascade with the organic host. Thus, acting as a bridge between the two components, IEICO facilitates cascading energy transfer of excitons and improves the charge dissociation at interface.

#### 4.2.6. SnS_2_

Tan (2011) obtained composite films using SnS_2_ QDs (5–7 nm in size) and MDMO-PPV for fabricating solar cells [221]. Additionally, in this case, a ligand exchange process was carried out for replacing oleylamine from the SnS_2_ QDs surface with pyridine for enhancing excitons splitting efficiency in the PV structure. The best cell efficiency (0.26%) was achieved for the structure containing an optimum SnS_2_ QDs amount (50%) being related to an annealing treatment applied for optimizing the interpenetrating network in order to enhance the electron transport through it.

#### 4.2.7. FeS_2_

Yu (2015) prepared composite films involving FeS_2_ QDs and P3HT for developing solar cells [173]. Again, a ligand exchange process was made for replacing octadecylamine from the FeS_2_ QDs surface with pyridine for increasing charge collection in the PV structure. The best cell efficiency (0.45%) reached for the structure containing an optimized P3HT:FeS_2_ QDs ratio (1:2) was linked to the electron acceptor role played by FeS_2_ QDs which leads to an improvement of the charge collection at the electrodes.

#### 4.2.8. ZnSe

Benchaabane (2017) integrated composites based on oleic acid capped-ZnSe nanoparticles (3–4 nm in size) and PVK in PV cells [222]. The influence of the oleic acid capped-ZnSe nanoparticles amount (varied from 10% to 90%) on the device performance was investigated. Thus, a 0.02% cell efficiency was determined for the structure based only on PVK while a 0.25% cell efficiency was recorded for that containing 90% oleic acid capped-ZnSe nanoparticles due to the enlarged polymer:nanoparticle interface area, which enhances the exciton dissociation probability.

#### 4.2.9. ZnS

Jabeen (2017) developed hybrid cells (in an inverted configuration) based on P3HT and ZnS QDs (8–10 nm in size) or Mn-doped ZnS QDs [223]. Thus, a cell efficiency of 0.48% (without annealing) and 0.52% (with annealing) was obtained for the device based on un-doped ZnS QDs, while a cell efficiency of 0.73% (without annealing) and 0.83% (with annealing) was reached by the device based on Mn-doped ZnS QDs. The cell efficiency was enhanced by a factor of 0.52 (without annealing) and 0.59 (with annealing) taking into account that the band gap of ZnS QDs can be tuned by doping it with manganese.

Han (2018) studied ternary solar cells based on InP core-ZnS shell (InP-ZnS) QDs (3.52 nm in size) and PTB7-Th:PC71BM in an inverted device structure involving ZnO or ethanedithiol (EDT)-modified ZnO as CBL [224]. The cell efficiency increased from 9.1% for the cell prepared without QDs and untreated ZnO layer to 10.2% for that prepared with InP-ZnS QDs and EDT-modified ZnO. The overlapping of the PTB7-Th absorption band and InP-ZnS QDs emission band enhanced the photocurrent generation. Additionally, the uniform morphology and the surface potential of the modified ZnO layer increased the electron transport and reduced the charge carrier recombination.

#### 4.2.10. Cu_2_S

Lakhotiya (2019) used ternary blends based on Cu_2_S nanocrystals with dumbbell shape (less than 20 nm in diameter and length), PTB7-Th and PCBM for obtaining active layers for PV cells with inverted geometry [225]. The Cu_2_S nanocrystals amount was varied for evaluating the influence of this parameter on the device performance. Thus, the cell efficiency increases from 6.96% for the OPV cell to 8.20% for the HPV cell with an optimum CuS_2_ nanocrystals amount (3%). This improvement was associated with the presence of Cu_2_S nanocrystals that favors the formation of electron cascade energy levels between PTB7-Th and PCBM which, in turn, limits the trap-assisted recombination.

**Figure 9:**
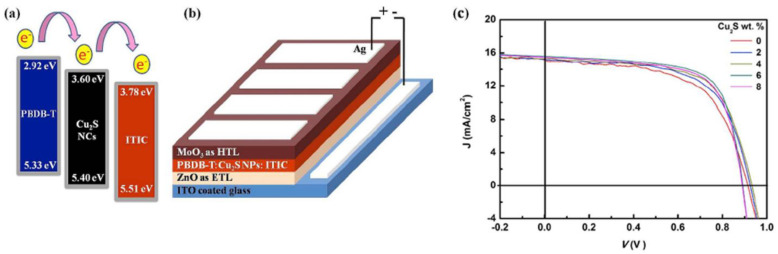
(**a**) Energy levels of PBDB-T, Cu_2_S and ITIC. (**b**) Schematic representation of the PV architecture. (**c**) J–V characteristics under illumination of the PV cells fabricated with hybrid films based on PBDB-T:Cu_2_S:ITIC blends with different weight ratios of Cu_2_S nanocrystals. Reprinted with permission from [226]. Copyright 2019 Elsevier.

Lakhotiya (2019) used ternary blends based on Cu_2_S nanocrystals with elongated shape (6–10 nm in diameter and 10–20 nm in length) and poly[(2,6-(4,8-bis(5-(2-ethylhexyl)thiophen-2-yl)-benzo[1,2-b:4,5-b′]dithiophene))-alt-(5,5-(1′,3′-di-2-thienyl-5′,7′-bis(2-ethylhexyl) benzo[1′,2′-c:4′,5′-c′]dithiophene-4,8-dione))] (PBDB-T) and ITIC for preparing active layers for PV cells having inverted configuration [226]. Once again, the influence of the Cu_2_S nanocrystals amount on the device performance was evaluated. Hence, the cell efficiency increases from 8.24% for the OPV cell to 9.53% for the HPV cell with an optimum CuS_2_ nanocrystals amount (6%). The enhancement was attributed to the presence of Cu_2_S nanocrystals, these acting as an electron cascade between PBDB-T and ITC enhancing the charge transfer and decreasing the trap-assisted recombination (Figure 9).

#### 4.2.11. CuS

Hamed (2019) added CuS nanoparticles (15–160 nm in size) in P3HT:PC61BM blends for developing PV devices, evaluating the influence of the inorganic nanoparticles on the solar cell performance [98]. Thus, a significant improvement was obtained when this *p*-type semiconductor was embedded in the active film, the cell efficiency increasing from 2.98% for the reference cell (without CuS particles) to 5.04% in the presence of an optimum CuS nanoparticles amount (3%). The improvement was related to the enhanced optical absorption assisted by a local surface plasmon resonance process as well as multiple light scattering on the inorganic nanoparticles within the active layer.

#### 4.2.12. CuInS_2_

Nam (2011) added CuInS_2_ QDs (~3 nm in size) within P3HT:PCBM blends for developing HPV devices [227]. For both organic compounds, the amounts were kept constant while that of CuInS_2_ QDs was varied for evaluating the influence of this parameter on the device performance. Thus, a cell efficiency of 2.44% was recorded for the reference cell based only on organic materials while a cell efficiency of 2.76% was achieved for the structure having a 1:1:0.2 weight ratio between the components. The enhancement was related to: an increase of the optical absorption due to the addition of QDs featured by a high absorption coefficient, an efficient charge carrier transfer favored by the adequate ordering of the energy levels and an enlargement of the interfacial area of the *p*-*n* junction due to the nanoscale composition.

Kumari (2014) used ternary blends based on CuInS_2_ QDs, P3HT and graphene for integrating them into PV cells [228]. The study has shown that the annealing temperature used for the treatment of the hybrid films plays a main role in the device performance. Hence, before annealing, the cell efficiency was 0.18% while, after annealing at an optimum temperature (120 °C), the cell efficiency reached 1.3%. The improvement was linked to the annealing process that leads to an increase of the interaction between the components which, in turn, leads to an enhancement of the absorption and exciton dissociation efficiency.

#### 4.2.13. PbS_x_Se_1-x_

Liu (2013) prepared hybrid layers based on PbS_x_Se_1-x_ nanocrystals (~7 nm in size) and PDTPBT, a low band gap polymer, in order to integrate them in the PV cells [229]. Additionally, a supplementary nanocrystals layer was inserted for enhancing the charge separation and transport and improving the device performance. Thus, the best cell efficiency (5.5%) is attributed to an optimum ratio between the polymer and inorganic nanocrystals (1:15) and to the formation of self-assembled vertical phase segregation in the blend layer, the vertical D-D:A-A structure significantly decreasing the charge recombination and increasing the charge dissociation and transport.

### 4.3. Silicon-Based Nanocomposites

Silicon nanostructures can be regarded as a viable and low-cost component that can be integrated in the hybrid solar cell devices taking into account that currently the PV industry is still mainly based on silicon. Although, the developed hybrid photovoltaic cells based on Si nanostructures involved mainly the stacked architecture in which an interpenetrating network is formed by disposing a polymer over a Si nanowire array (the inorganic nanoparticles being used to improve the light absorption, exciton dissociation and the carriers transport), some studies reported on the incorporation of the Si nanostructures in various organic compounds [230,231].

Eisenhawer (2011) investigated the properties of hybrid solar cells based on P3HT:PCBM and Si nanowires [232]. The study reveals that a cell efficiency of 4% is recorded using a low density of Si nanowires in the active layer, this being an improvement of 10% relative to the reference cell (without Si nanowires).

Svrcek (2011) obtained surfactant-free Si nanocrystals by employing laser ablation in water and electrochemical etching techniques [233]. The films based on P3HT and Si nanocrystals are integrated into HPV structures featured by an improvement of the photovoltaic properties due to an increase of the bulk heterojunction surface area resulting in an increase in the charge transport.

Svrcek (2012) developed also hybrid PV cells based on PTB7 and Si nanocrystals [234]. This time, a microplasma treatment was applied to the previously prepared Si nanocrystals for enhancing the electronic interactions with the polymer and increasing in this way the cell efficiency. Although this process seems to improve the cell efficiency, a lower value (0.03%) was recorded for this structure.

Dkhil (2012) prepared composite layers based on Si nanowires (less than 20 nm in diameter and ~10 µm in length) and MEH-PPV or PVK as polymer matrix, evaluating the influence of the polymer type on the properties of the fabricated structures [235]. Thus, better electrical parameters were recorded for the structure involving PVK (Pmax = 0.41 × 10^−7^ W) compared to the structure using MEH-PPV (Pmax = 0.47 × 10^−8^ W), in the last case a pronounced aggregation effect of the Si nanowires being remarked.

Liu (2012) fabricated the PV cells involving BHJ based on MDMO-PPV and Si nanocrystals (3–5 nm in size), evaluating the influence of the inorganic nanostructures amount on the cell performance [236]. Even if an improvement in the open circuit voltage was achieved relative to other cells prepared with P3HT, the recorded efficiency was only 0.49%.

Kim (2012) attached octanoic acid (OA) molecules on the surface of the Si nanoparticles (20 nm in size) for improving their dispersion in the BHJ films containing P3HT and PC61BM [237]. Indeed, an increase in the cell efficiency (2.7%) was recorded for the HPV prepared with the composite films due to a better dispersion of the OA-attached Si nanoparticles within the obtained film.

Kim (2012) analyzed also the properties of the PV cell structures prepared with Si nanocrystals and P3HT:PCBM blend [101]. It was emphasized that the presence of the inorganic nanoparticles in the active layer induced the coupling of optical excitation between the polymers and Si nanocrystals, the resulted extended excitation response improving the electrical performance of the cells, an efficiency of 3.2% being recorded.

Ding (2013) fabricated PV cells based on hybrid layers containing P3HT and Si nanocrystals (6 nm in size), evaluating the dependence between the concentration of the inorganic nanostructures and device performance [238]. The study revealed that the concentration of the Si nanocrystals strongly influences the cell efficiency, the best value (0.15%) being attributed mainly to the increase of the exciton dissociation due to a larger interface between P3HT and Si nanocrystals.

Yan (2013) embedded Si nanoparticles (~3 nm in size) in a MEH-PPV:PCBM active layer [239]. The enhancement in the cell efficiency (2.28%) was related to a better charge generation at the interface formed between the polymer and inorganic nanostructures.

Chang (2013) doped P3HT:PCBM blends with Si nanoparticles (80–100 nm in size) in order to obtain active layers for PV devices [240]. The Si nanoparticles enhances the device performances (3.38% efficiency) contributing to the excitons separation and assuring electron pathways in the formed structures.

Dkhil (2014) analyzed composite films based on P3HT and Si nanowires (less than 40 nm in diameter and ~10 µm in length) for developing PV cells [174]. The influence of the Si nanowires concentration (in relation with the P3HT amount) and the solvent type (tetrahydrofuran, ortho-dichlorobenzene) used in the deposition of the active film were investigated. The results emphasized that the photoluminescence quenching and the cell performance depend on the film morphology which, in turn, is strongly influenced by the solvent used in the deposition of the active layer. Therefore, a suitable solvent (tetrahydrofuran) and an optimum Si nanowires amount (15%) can provide an appropriate mixing between the P3HT and inorganic nanostructures, this being responsible for the formation of a network in the hybrid layer which leads to the improvement of the cell efficiency, the best recorded value being 0.08%.

Yan (2014) grafted amino groups on the surface of the Si nanoparticle (~2 nm in size) for improving their dispersion in water, further these stable functionalized Si nanoparticles were incorporated in a HBL layer based on PEDOT:PSS and finally the obtained layer was integrated in a cell structures having MEH-PPV:PCBM blend as active layer [97]. Hence, the cell efficiency increased from 2.24% for the structure containing a simple PEDOT:PSS layer to 2.46% for the cell prepared with the hybrid buffer layer, the enhancement being correlated with the increase of the PEDOT:PSS conductivity in the HBL layer. Uniformly dispersed in the PEDOT:PSS solution, the amino-grafted Si nanoparticles are responsible for the formation of the morphology with small-size domains in the PEDOT:PSS films. Furthermore, acting as screens between the PEDOT conducting polymer and PSS ionomers, the amino-grafted Si nanoparticles increase the charge transport in the HBL.

Chehata (2015) fabricated composite films containing MEH-PPV and Si nanowires (~100 nm in diameter and ~16 µm in length) functionalized with polystyrene [241]. Hence, the intrinsic surface defects of the Si nanowires are passivated by the surface functionalization with polystyrene. In this way, the interface between the MEH-PPV and Si nanowires is improved and the device performance increased. In the cell based on the film having 1:4 ratio between the two components, the MEH-PPV photoluminescence decreases and the generated excitons dissociate, an efficiency of 0.03073% being recorded.

Ding (2016) reported on the fabrication of HPV cells based on hybrid films containing Si nanocrystals (6 nm in size), PTB7 and PC71BM, two individual junctions being designed and arranged in parallel with each other in order to form a double-parallel-junction [231]. Thus, a BHJ based on PTB7 and Si nanocrystals is arranged in parallel with a bilayer heterojunction based on PTB7 and PC71BM. The cell efficiency reached at 2.46% being linked to the enhancement of the absorption in a wide energy domain due to the formed double parallel junctions. Hence, PC71BM can extend light absorption of Si nanocrystals and PTB7, complementary absorption being obtained by these two parallel sub-junctions. In this way, the device efficiency can be enhanced more than twice in comparison to the reference cell based only on a single junction.

Arunmetha (2018) investigated PV structures based on films containing P3HT and Si nanoparticles (30–50 nm in size) [242]. The cell efficiency is improved from 0.099% for the reference cell based only on the organic compound to 1.067% for the cell using Si nanoparticles, the efficiency increase being due to the contribution of the inorganic nanoparticles in the absorption from the ultraviolet and visible regions.

**Figure 10:**
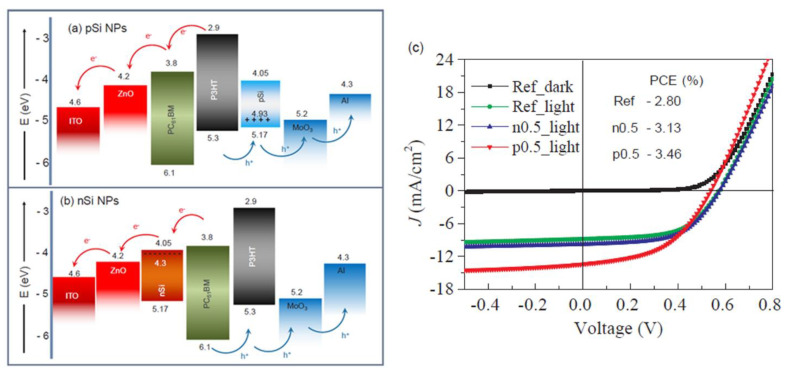
Energy band alignment in the devices developed with hybrid films based on P3HT:PC61BM blends and (**a**) *p*-Si nanoparticles or (**b**) *n*-Si nanoparticles. (**c**) J–V curves for the reference, *n*-Si and *p*-Si nanoparticles incorporated devices and their photo-conversion efficiency. Reprinted with permission from [104]. Copyright 2018 Elsevier.

Hemaprabha (2018) developed PV cells based on composite films containing P3HT:PC61BM and Si nanoparticles (~60 nm in size) that can act as *n*-type material (phosphorous doped silicon) or *p*-type material (boron doped silicon) [104]. Hence, by doping, the Fermi levels of the Si nanoparticles can be tuned, the cascading energy levels being responsible for the improvement in the device performance: the cell efficiency increased from 2.8% to 3.13% when *n*-type Si nanoparticles are used while the device efficiency reaches to 3.46% when *p*-type Si nanoparticles are involved (Figure 10).

Vinoth (2019) fabricated hybrid cells based on P3HT and Si nanoparticles (~30 nm in size) and evaluated the influence of the Ca/Al electrode thickness on their performance [243]. Thus, the cell efficiency increases from 3.06% for the cell having only an Al electrode (100 nm in thickness) at 3.77% for the cell involving a Ca/Al electrode (10 nm/90 nm in thickness). The improvement was related to the presence of the Ca interface layer which leads to higher charge extraction from the device and a subsequent decrease in the charge recombination.

### 4.4. Power Conversion Efficiency of the Analyzed PV Cells Fabricated with Hybrid Composite Layers–Summary

It can be concluded that starting from OPV structures, various HPV cells containing BHJ based on composite materials and OPV cells containing additional buffer layers (ABL or CBL) based on hybrid films were developed. BHJ can be based on organic:inorganic composites in different combinations: (i) An organic *p*-type compound and an inorganic *n*-type semiconductor (e.g., P3HT:ZnO); (ii) *p*-*n* organic compounds and an inorganic *n*-type or *p*-type semiconductor (e.g., P3HT:PCBM:ZnO or P3HT:PCBM:CuO); and (iii) *p*-*n* organic compounds and *p*-*n* inorganic semiconductor nanostructures (e.g., P3HT:PCBM:ZnO:CuO). Therefore, in Table 2 are summarized the fabricated PV devices based on hybrid composite layers in both architectures (regular or inverted), the hybrid film acting as active layer or as additional buffer layer and their PCE values. Additionally, the PCE values of the PV devices fabricated in the absence of the inorganic nanostructures are also given, these structures being regarded as reference relative to the investigated PV devices based on hybrid composite layers.

## 5. Hybrid Nanocomposite Films Deposited by MAPLE for PV Cells

MAPLE revealed its potential for the development of hybrid layers based on polymer and inorganic nanostructures for applications in the photovoltaic field. Further, the few studies focused on this research topic will be summarized.

Ge (2015) prepared nanocomposite layers based on CdSe nanoparticles (~7–9 nm in size) and PCPDTBT (a low band gap polymer) by emulsion-based RIR-MAPLE [65]. The influence of the ligand type (trioctylphosphine oxide (TOPO), pyridine), various solvents (chlorobenzene, dichlorobenzene, trichlorobenzene), and two deposition techniques (RIR-MAPLE and the common spin-casting) on the morphology of the blended films was analyzed. A ligand exchange process was applied for replacing the insulating TOPO from the CdSe nanoparticles with pyridine in order to improve the charge transfer between the polymer (electron donor) and CdSe nanoparticles (electron acceptor). CdSe nanoparticles functionalized with TOPO or pyridine were added in various amounts (60–90% in respect with the amount of polymer) into PCPDTBT (5 mg/mL concentration) for obtaining hybrid active films by RIR-MAPLE and spin-coating. In the case of RIR-MAPLE deposition, a small amount of sodium dodecyl sulfate (SDS) surfactant (0.001 wt%) was also introduced to stabilize the CdSe nanoparticle target emulsion.

**Figure 11:**
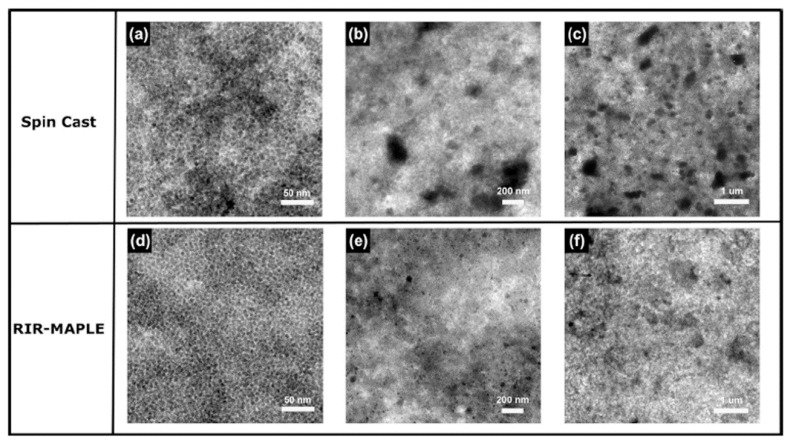
TEM images of PCPDTBT:pyridine-capped CdSe blends deposited by (**a**–**c**) spin-casting using trichlorobenzene as solvent and (**d**–**f**) RIR-MAPLE using trichlorobenzene as primary solvent. Reprinted with permission from [65]. Copyright 2015 Elsevier.

The morphological investigations have shown that the films deposited by RIR-MAPLE are continuous, uniform and smooth (Figure 11) being characterized by a low roughness. It has to be mentioned that some smaller clusters can be noted in the films deposited by RIR-MAPLE while larger aggregates can be easily observed as black clusters in the films deposited by spin-casting. Thus, the tendency of CdSe nanoparticles to form aggregates which further lead to polymer and nanoparticle phase segregation is minimized when RIR-MAPLE is used as the deposition technique. Furthermore, the optical investigations carried out on both TOPO-capped CdSe nanoparticles and pyridine-capped CdSe nanoparticles, before (in solution) and after RIR-MAPLE deposition demonstrated that the optical properties of the inorganic nanoparticles are preserved in the films obtained by this laser technique.

**Figure 12:**
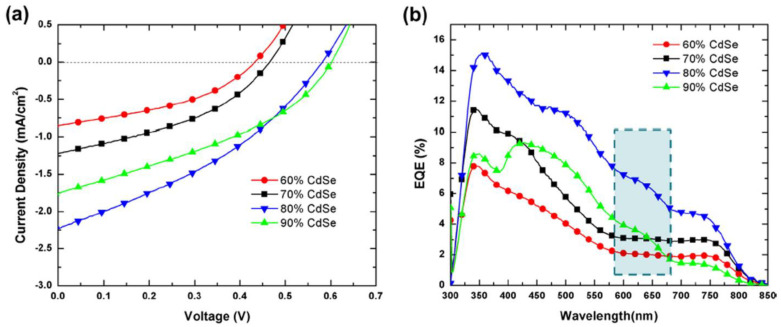
(**a**) J–V curves of PV cells developed on hybrid layers based on PCPDTBT:pyridine-capped CdSe blends with different CdSe loading, the films being deposited by RIR-MAPLE. (**b**) EQE of the corresponding solar cells. Reprinted with permission from [65]. Copyright 2015 Elsevier.

The PV structures were fabricated in a regular configuration involving hybrid films and a supplementary PEDOT:PSS layer. In the case of PV cell containing films deposited by RIR-MAPLE, the best cell efficiency (0.41%) was achieved for the structure containing 80% CdSe nanoparticle loading (Figure 12).

Even though, the cell efficiency is low (0.4%) due to the presence of SDS added to stabilize the CdSe nanoparticle during the preparation of the RIR-MAPLE target, the involvement of other surfactant type could improve the performance of such PV devices fabricated with layers deposited by RIR-MAPLE technique. Therefore, this study can be regarded as a step forward in the field of nanocomposite films fabricated by an alternative deposition technique.

Recently, Socol (2020) used metal phthalocyanines (ZnPc, CoPc) and ZnO nanoparticles for developing hybrid layers for PV applications [44,64]. Thus, composite films based on a binary mixture (ZnPc and ZnO nanoparticles having ~30 nm in size) [44] or a ternary mixture (CoPc, C60 and ZnO nanoparticles with ~20 nm in size) [64] were deposited by MAPLE at a moderate laser fluence (350 mJ/cm^2^ for ZnPc:ZnO blend and 240 mJ/cm^2^ for CoPc:C60:ZnO blend), in both cases dimethyl sulfoxide being involved as the solvent in the preparation of the MAPLE target. The metal phthalocyanines concentration was kept constant (3% *w*/*v*) while the ZnO nanoparticles concentration (*w*/*w* ratio) was varied (P1 (1:0.15), P2 (1:0.35) and P3 (1:0.55) for ZnPc:ZnO mixture and P0 (1:1:0), P1 (1:1:0.25), P2 (1:1:0.75), and P3 (1:1:1) for CoPc:C60:ZnO mixture) in order to evaluate the influence of this parameter on the device performance. Additionally, C_60_ fullerene (in the same amount with the CoPc) was added for improving the exciton dissociation at interface [244].

**Figure 13:**
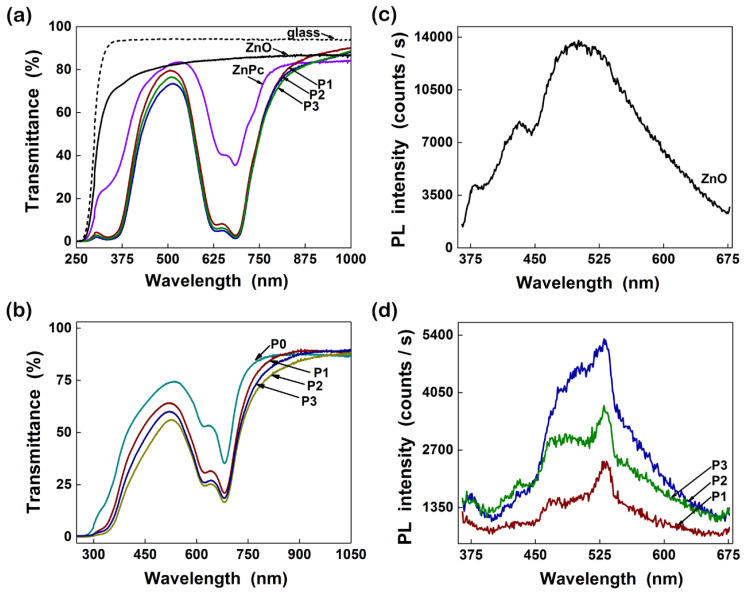
Optical properties, (**a**,**b**) transmittance and (**c**,**d**) photoluminescence, of MAPLE deposited hybrid films based on ZnPc:ZnO (**a**,d) or CoPc:C60:ZnO (**b**) in both hybrid type being varied the amount of ZnO nanoparticles: 1:0.15 (P1), 1:0.35 (P2) and 1:0.55 (P3) for blends based on ZnPc (**a**,**d**) and 1:1:0 (P0), 1:1:0.25 (P1), 1:1:0.75 (P2), and 1:1:1 (P3) for blends based on CoPc (**b**). The emission spectrum of film based on ZnO nanoparticles deposited by MAPLE (**c**). (**a**,**c**,**d**) reprinted with permission from [44]. Copyright 2020 Elsevier. (**b**) reprinted from [64].

The optical investigations carried on hybrid films revealed that the UV-VIS spectra are dominated by the absorption bands associated to the metal phthalocyanines (ZnPc and CoPc) while in the photoluminescence spectra are identified the emission bands attributed to the ZnO (Figure 13). Thus, the UV-VIS spectra of the hybrid films evidence the absorptions bands characteristic to the donor material, the B-Soret band and the Q band with two minima [135]. The absorption specific to ZnO (the small shoulder at ~380 nm [245]) was identified in the layer deposited from the target containing only ZnO while in the composite films this absorption is masked by the strong absorption of the metal phthalocyanine. The photoluminescence spectra of the hybrid films disclose the emission bands specific to ZnO and a quenching effect related to the charge transfer to the metal phthalocyanine [246].

**Figure 14:**
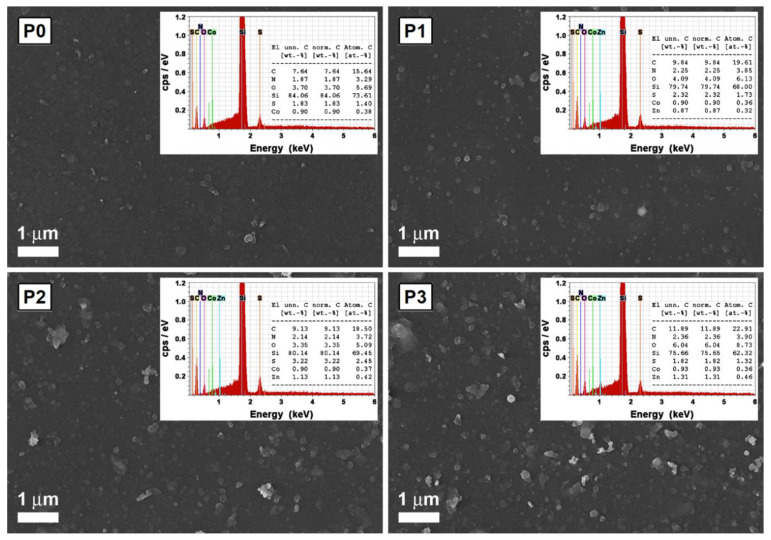
FESEM images of the MAPLE deposited thin films based on CoPc:C60:ZnO blends with various amount of ZnO nanoparticles: 1:1:0 (**P0**), 1:1:0.25 (**P1**), 1:1:0.75 (**P2**), and 1:1:1 (**P3**). Insets: EDX spectra and weight and atomic percentages of the elements in the corresponding samples. Reprinted from [64].

The morphological analysis shown a clusterization process as the ZnO amount increases in the hybrid films, the stoichiometric transfer of the source material being confirmed by the EDX data (Figure 14).

**Figure 15:**
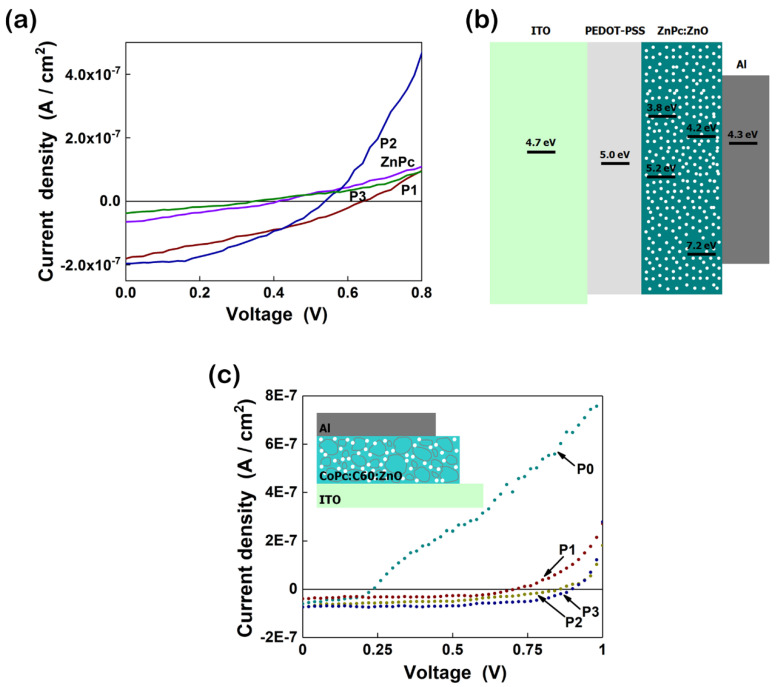
J-V characteristics of the structures developed on the MAPLE deposited layers based on (**a**) ZnPc:ZnO or (**c**) CoPc:C60:ZnO blends, in both hybrid type being varied the amount of ZnO nanoparticles: 1:0.15 (P1), 1:0.35 (P2) and 1:0.55 (P3) for blends based on ZnPc (**a**) and 1:1:0 (P0), 1:1:0.25 (P1), 1:1:0.75 (P2), and 1:1:1 (P3) for blends based on CoPc (**c**). Schematic representation of the devices involving MAPLE deposited layers based on ZnPc:ZnO (**b**) or CoPc:C60:ZnO (inset **c**). (**a**,**b**) reprinted with permission from [44]. Copyright 2020 Elsevier. (**c**) reprinted from [64].

The electrical investigations made on PV structures developed, in regular configuration, with the MAPLE layers emphasized that the addition of ZnO in the organic active layer improved the J_SC_, 1.97 × 10^−7^ A/cm^2^ for ZnPc:ZnO blend and 7.5 × 10^−8^ A/cm^2^ for CoPc:C60:ZnO in comparison with those recorded on the structures without ZnO, 6.41 × 10^−8^ A/cm^2^ for ZnPc, and 5.9x10^−8^ A/cm^2^ for CoPc:C60, respectively (Figure 15).

Although the electrical performances are not impressive, by carrying systematic studies, new information regarding the deposition parameters (solvents, concentration of organic and inorganic components, inorganic particle size and shape, laser fluence, target-substrate-distance, etc.) can be acquired which could be very useful in enhancing the device performance. For example, in the case of OPV cells involving P3HT:PC61BM or PCPDTBT:PC71BM layers deposited by emulsion-based RIR-MAPLE, by analyzing the deposition from six different solvents, Ge succeeded to enhance the efficiency of the PV cells based on P3HT:PC61BM blends from 1.84% to 3.27% using 1,2,4-trichlorobenzene instead of toluene [62].

## 6. Conclusions and Future Perspectives

In this review, we have highlighted the recent advances regarding the development of PV cells based on hybrid composite thin films deposited by spin-coating, the most common deposition approach, and MAPLE, a relatively new deposition process. Thus, we summarize the organic compounds (conducting polymers and metal phthalocyanines as *p*-type materials and fullerene derivatives and non-fullerene compounds as *n*-type materials) and inorganic semiconductor nanostructures (metal oxides, chalcogenides, and silicon) that are most frequently used in the preparation of the organic:inorganic mixtures in order to obtain hybrid layers for PV devices. The information from this overview can be useful for the research carried out in the solar cell area regarding the enhancement of the PV cells’ performance through the design of hybrid composite films with adequate properties by controlling various factors (type of the organic and inorganic components, solvent type, organic and inorganic component concentrations, size and shape of the inorganic nanostructures, experimental parameters involved in each deposition technique, and so on).

In the last decade, many strategies have been elaborated in order to increase PV cell efficiency based on structures employing hybrid layers deposited by spin-coating technique. Thus, various PV cell structures, in regular or inverted configuration, involving one or two organic compounds paired with one or two inorganic semiconductor nanoparticles, with or without additional buffer layers, were fabricated, and complexly characterized for evaluating the influence of different factors on the electrical parameters of the PV cells. Based on the information presented above, the following aspects must be taken into consideration for enhancing the device performance: (i) The addition of an optimum amount of inorganic nanostructures in the active layer for enhancing the light absorption (probably the most used approach for improving the cell efficiency), a small quantity of inorganic compound being insufficient to provide a large interface for the exciton dissociation while a high quantity of inorganic nanoparticles can lead to an agglomeration process which influences the charge carrier transport; (ii) the involvement of low band gap polymers, such as PTB7 or its derivative PTB7-Th, paired with metal oxides (TiO_x_ [91], ZnO [96], Cr_2_O_3_ [189]), chalcogenides (CdSe [211], CdS [121], InP-ZnS [224], Cu_2_S [225]) or silicon [231]; (iii) the use, in the CBL layer, of PEG (polymer with lone electron pairs of oxygen in its backbone) or F4-TCNQ (a compound with cyano groups) for filling the metal oxide defects (ZnO [96,176], TiO_x_ [91]), avoiding the trap-assisted recombination loss and allowing an efficient charge transport; (iv) applying a proper annealing treatment for improving the crystallinity of the organic polymers, like P3HT [214] or MEHPPV [133], used paired with CdTe; (v) the use of an appropriate ratio between the organic and inorganic components and a suitable solvent for both components for improving their compatibility and obtaining a homogeneous dispersion of the acceptor material in the mixture blend in order to generate an optimal nano-phase separation of the components inside the active film and to assure percolation pathways for the charge carriers through the electrodes (MEHPPV:CdTe [133]); (vi) the addition of non-fullerene acceptor materials featured by a strong absorption and high electron mobility, like ITIC, in blends based on conjugated polymers, such as PTBT-Th [96] or PBDB-T [226], for facilitating the intramolecular charge transfer and to improve the light absorption by broadening the absorption spectrum; (vii) the use of small molecules, such as IEICO, characterized by complementary absorption to inorganic nanocrystals, like PbS QDs [220], which can act as a bridge between the components facilitating the energy transfer of excitons in cascade and improving the charge dissociation at interface; (viii) the replacement, by a ligand exchange process, of the insulating ligands from the surface of the inorganic colloidal nanostructures (originating from their synthesis) with smaller molecules (examples: thiols or uree for CdSe QDs [202,204]), iodine for PbS [125], pyridine, amines, etc.) more suitable in the charge transfer; (ix) the morphology of the inorganic nanostructures, due to their aspect ratio, those having an elongated shape (CdSe [200,203], CdS [121], Cu_2_S [226]) can provide a continuous pathway for the charge transport process; (x) the size of the inorganic colloidal nanostructures (especially in the case of chalcogenides nanocrystals) related to the quantum confinement effects; (xi) the development of inverted device architecture instead of the regular architecture, the photodegradation of the organic compound from the active layer being avoided in the inverted structure by the presence of a ZnO layer that can act as a barrier for UV radiation [195,226]. Thus, at the nanoscale level, in the donor-acceptor layer, a good control for preventing the exciton recombination and optimizing the alignment of the energy levels at the organic:inorganic interface is required.

It must be highlighted that by using inorganic colloidal nanostructures synthesized by aqueous solution-based routes and conjugated polymers prepared from water-soluble precursors, an aqueous solution-processed hybrid solar cells can be fabricated by more environmentally friendly routes in comparison with those frequently used for the devices obtained from organic soluble materials.

Recently, the potential of a relatively new deposition technique, MAPLE, was evaluated for preparing hybrid layers based on PCPDTBT:CdSe QDs blends [65] and metal phtalocyanine:ZnO nanoparticle blends [44,64] for PV cells. Although the electrical performances are not impressive, the device performance can be enhanced by optimizing the parameters involved in the MAPLE deposition. Moreover, MAPLE can be regarded as a promising approach in the PV area taking into account the ability to prepare thin films using the same solvent (the deposition of a second layer can be made without affecting the first deposited layer) and the facility to deposit thin films on plastics, which is very attractive in the area of flexible electronics.

In the opinion of the authors, in the near future, further improvements in the performance of PV cells based on composite layers will be reached taking into account the “library” of the organic materials and of the inorganic nanostructures that can be used for developing PV cell structures. Therefore, the enhancement of hybrid solar cells’ performance can be reached by tailoring the optical and electrical properties of these components through their particular features (e.g., designed D-A conjugated polymers, metal oxide nanoparticles with specific size and morphology, chalcogenide colloidal nanocrystals characterized by quantum confinement effects, etc.). Additionally, the increase in the PV cell efficiency can be achieved by combining the inorganic nanoparticles (with complementary light absorption spectra) with a *p*-*n* organic material blend instead of a single *p*-type organic material. By using inorganic nanostructures with different morphologies (e.g., rods, spherical particles, etc.), the device efficiency can be also enhanced due to a better connection between these structures and the polymer matrix that can facilitate the electron transport and reduce the carrier recombination. Furthermore, the permanent demand to develop PV cells on rigid or flexible substrates by environmentally responsible technologies is still a major challenge. Consequently, the full potential of the hybrid nanocomposite layers in the solar cell applications will be realized only when these layers are prepared by more environmentally friendly approaches.

## Figures and Tables

**Table 1:** Schematic illustration of some OPV and HPV cell architectures in correlation with the organic and inorganic materials used in their development (based on the data from the literature review).

**Regular PV Cell Architectures**		
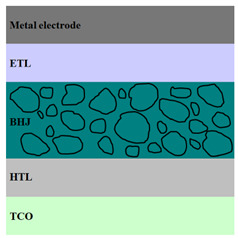	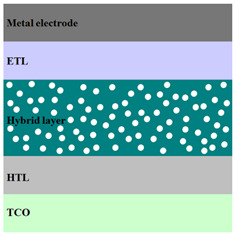	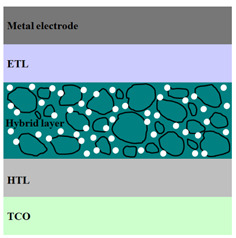
ITO/PEDOT:PSS/ P3HT:PCBM/LiF/Al [98]	ITO/PEDOT:PSS/ P3HT:CdSe/LiF/Al [99]	ITO/PEDOT:PSS/ P3HT:PCBM:CuS/LiF/Al [98]
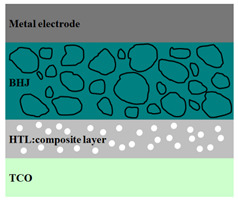	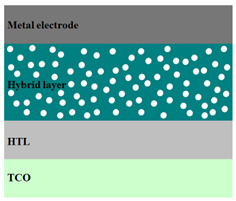	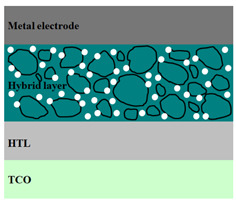
ITO/PEDOT:PSS:Si/ MEH-PPV:PCBM/Al [97]	ITO/PEDOT:PSS/ P3HT:ZnO/Al [100]	ITO/PEDOT:PSS/ P3HT:PCBM:Si/Al [101]
**Inverted PV cell architectures**		
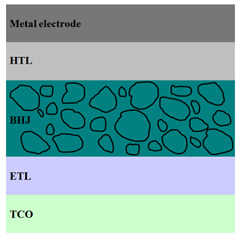	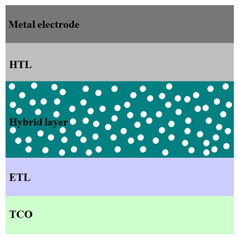	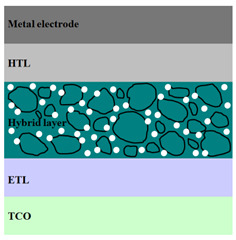
ITO/ZnO/ P3HT:PCBM/MoO_3_/Ag [94]	ITO/ZnO/ P3HT:ZnO/PEDOT:PSS/Au [102]	ITO/ZnO/ P3HT:PCBM:ZnO/MoO_3_/Ag [94]
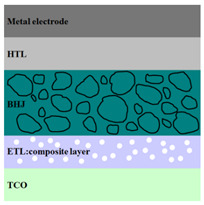	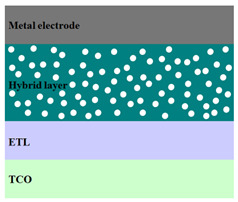	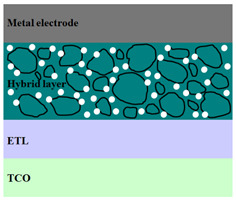
ITO/F4-TCNQ:ZnO/ PTB7-Th:PC71BM/MoO_3_/Al [96]	ITO/TiO_2_/ MEH-PPV:CdS/Au [103]	ITO/ZnO/ P3HT:PCBM:Si/Al [104]

**Table 2:** PV cell structures fabricated with hybrid composite layers, device architectures, the role played by the hybrid layers: active layer (AL), cathode buffer layer (CBL), or anode buffer layer (ABL), PV cell efficiency, and the year when the work was reported (based on the data from the literature review). For comparison reason, the efficiency of the reference PV cell (without the hybrid layer) is also given if the published data were available.

PV Cell Structure	Device Architecture	Composite Layer Role	PCE (%)	PCE (%) Reference PV Cell	Published Year	Ref.
**Composites based on ZnO**						
ITO/PEDOT:PSS/P3HT:ZnO/Al	Regular	AL	1.7	-	2011	[100]
ITO/PEDOT:PSS/P3HT-E:ZnO/Al			0.83	-		
ITO/ZnO/P3HT:ZnO/PEDOT:PSS/Au	Inverted	AL	1	-	2011	[102]
ITO/PEDOT:PSS/P3HT:ZnO/Al	Regular	AL	0.12	-	2012	[175]
ITO/PEDOT:PSS/P3HT:CdS-ZnO/Al			0.38	-		
ITO/PEG:ZnO/P3HT:PCBM/PEDOT:PSS/Ag	Inverted	CBL	3.3	2.3	2013	[176]
ITO/PEDOT:PSS/P3HT:ZnO/Al	Regular	AL	0.21	-	2014	[177]
ITO/PEDOT:PSS/P3HT:PCBM:ZnO/Al	Regular	AL	0.77	0.65	2015	[178]
ITO/TiO_2_/P3HT:PCBM:ZnO/Al	Inverted		0.63	0.61		
ITO/ZnO/P3HT:PCBM:ZnO/MoO_3_/Ag	Inverted	AL	2.96	2.69	2015	[94]
ITO/TiO_2_/P3HT:PCBM:ZnO/MoO_3_/Al	Inverted	AL	1.7	1	2016	[179]
ITO/F4-TCNQ-ZnO/PTB7-Th:PC71BM/MoO_3_/Al	Inverted	CBL	8.14	7.17	2020	[96]
ITO/F4-TCNQ-ZnO/PTB7-Th:ITIC/MoO_3_/Al			6.96	6.13		
**Composites based on CuO**						
ITO/PEDOT:PSS/P3HT:PC70BM:CuO/Al	Regular	AL	2.963	2.106	2015	[180]
ITO/ZnO/P3HT:PCBM:CuO/MoO_3_/Ag	Inverted	AL	3.7	3.4	2015	[181]
ITO/ZnO/P3HT:PCBM:ZnO:CuO/MoO_3_/Ag	Inverted	AL	4.09	3.45	2015	[182]
ITO/PEDOT:PSS/P3HT:PC70BM:CuO/ZnO/Al	Regular	AL	3.95	2.358	2016	[183]
ITO/ZnO/P3HT:PCBM:CuO/MoO_3_/Ag	Inverted	AL	4.1	3	2019	[184]
ITO/PEDOT:PSS/P3HT:PC70BM:CuO/Al	Regular	AL	3.82	2.85	2020	[185]
**Composites based on TiO_2_ or TiO_x_**						
ITO/PPV/PPV:TiO_2_/LiF/Al	Regular	AL	0.11	-	2012	[186]
ITO/PEDOT/P3HT:PCBM:TiO_2_/LiF/Al	Regular	AL	2.1	1.6	2015	[187]
ITO/ZnO/P3HT:PCBM:TiO_2_/MoO_3_/Ag	Inverted	AL	3.10	2.69	2015	[94]
ITO/ZnO/P3HT:PCBM:ZnO:TiO_2_/MoO_3_/Ag	Inverted		3.03	2.69		
ITO/PEDOT:PSS/CSA-doped PANI-TiO_2_/Al	Regular	AL	0.21	-	2016	[188]
ITO/PEDOT:PSS/PEG-TiO_x_/ PDTTBT-3:PC71BM/MoO_3_/Ag	Inverted	CBL	5.14	2.80	2015	[91]
ITO/PEDOT:PSS/PEG-TiO_x_/ PTB7:PC71BM/MoO_3_/Ag			8.72	6.82		
ITO/PEDOT:PSS/PEG-TiO_x_/ PTB7-Th:PC71BM/MoO_3_/Ag			9.05	7.36		
**Composites based on Cr_2_O_3_**						
ITO/ZnO/P3HT:PCBM:Cr_2_O_3_/MoO_3_/Ag	Inverted	AL	3.67	2.51	2018	[189]
ITO/ZnO/PTB7:PC70BM:Cr_2_O_3_/MoO_3_/Ag			6.72	5.61		
**Composites based on CdSe**						
ITO/PEDOT:PSS/P3HT:CdSe/Al	Regular	AL	0.45	-	2011	[194]
ITO/PEDOT:PSS/P3HT:CdSe/LiF/Al	Regular	AL	1.12	-	2011	[99]
ITO/PEDOT:PSS/P3HT:CdSe/ZnO/Al	Regular	AL	2.2	-	2011	[195]
ITO/PEDOT:PSS/P3HT:CdSe/Al	Regular	AL	2.0	-	2011	[196]
ITO/PEDOT:PSS/PCPDTBT:CdSe/Al	Regular	AL	2.7	-	2011	[197]
ITO/PEDOT:PSS/P3HT:CdSe/Al	Regular	AL	1.9	-	2012	[198]
ITO/PEDOT:PSS/P3HT:CdSe/Al	Regular	AL	2.17	-	2012	[199]
ITO/PEDOT:PSS/PCDTBT:CdSe/Al			2	-		
ITO/PEDOT:PSS/PCPDTBT:CdSe/Ca/Ag	Regular	AL	2.8	-	2012	[200]
ITO/PEDOT:PSS/PCPDTBT:CdSe/Ca/Ag			3.6	-		
ITO/PEDOT:PSS/P3HT-b-PDMAEMA:CdSe/Al	Regular	AL	0.1	-	2012	[201]
ITO/PEDOT:PSS/P3HT:CdSe-SeU/Al	Regular	AL	2.63	-	2013	[202]
ITO/PEDOT:PSS/PCPDTBT:CdSe/Al	Regular	AL	4.7	-	2013	[203]
ITO/PEDOT:PSS/PCPDTBT:CdSe/CdSe/PFN/Al	Regular	AL	4.18	-	2014	[204]
ITO/P3OT:CdSe/Al	Regular	AL	0.51	0.025	2016	[205]
ITO/TiO_2_/P3HT:CdSe/MoO_3_/Au/Ag	Inverted	AL	1.7	-	2017	[207]
ITO/PEDOT:PSS/PCPDTBT:CdSe/Al	Regular	AL	2.81	-	2019	[208]
FTO/TiO_x_/P3HT:PCBM:CdSe/MoO_3_/Ag	Inverted	AL	2.55	1.99	2020	[209]
ITO/ZnO/PCDTBT:PCBM:CdSe/MoO_3_/Ag	Inverted	AL	5.02	3.62	2020	[210]
ITO/ZnO/PTB7-Th:PC71BM:CdSe/MoO_3_/Ag	Inverted	AL	9.57	8.58	2021	[211]
**Composites based on CdTe**						
ITO/PEDOT:PSS/PNV:CdTe/Al	Regular	AL	0.86	0.001	2011	[212]
ITO/TiO_2_/CdTe/CdCl_2_/PPV:CdTe/MoO_3_/Au	Inverted	AL	4.76	-	2013	[213]
ITO/TiO_2_/CdTe/P3HT:CdTe/MoO_3_/Au	Inverted	AL	4.32	-	2015	[214]
ITO/TiO_2_/CdTe/MEHPPV:CdTe/MoO_3_/Au	Inverted	AL	4.2	-	2016	[133]
ITO/TiO_2_/TiO_2_:CdTe/CdTe:PPV/MoO_3_/Au	Inverted	AL	6.01	-	2017	[215]
ITO/ZnO/ZnO:CdTe/CdTe/CdTe:PPV/MoO_3_/Au	Inverted	AL	6.51	-	2019	[216]
ITO/TiO_2_/TiO_2_:CdTe/CdTe/CdTe:PPV/MoO_3_/Au			5.87	-		
**Composites based on CdS**						
ITO/TiO_2_/MEH-PPV:CdS/Au	Inverted	AL	0.23	0.1	2011	[103]
ITO/ZnO/PTB7:CdS:PCBM/MoO_3_/Ag	Regular	AL	7.01	6.3	2016	[121]
FTO/TiO_2_/P3HT:PCBM:CdS/MoO_3_/Ag	Inverted	AL	2.42	1.2	2021	[217]
**Composites based on PbSe or PbS**						
ITO/PEDOT:PSS/P3HT/P3HT:PbSe/Al	Regular	AL	0.26	0.1	2011	[218]
ITO/PEDOT:PSS/Si-PCPDTBT:PbS/ZnO/Al	Regular	AL	4.8	-	2016	[125]
ITO/ZnO/PBDTTT-E-T:IEICO:PbS/MoO_3_/Ag	Inverted	AL	13.1	-	2019	[220]
**Composites based on SnS_2_ or FeS_2_**						
ITO/PEDOT:PSS/MEHPPV:SnS_2_/ZnO/Al	Regular	AL	0.26	-	2011	[221]
ITO/PEDOT:PSS/P3HT:FeS_2_/ZnO/Al	Regular	AL	0.61	-	2015	[173]
**Composites based on ZnSe or ZnS**						
ITO/PVK:OA-ZnSe/Al	Regular	AL	0.25	0.02	2017	[222]
ITO/ZnO/P3HT:ZnS/MoO_3_/Ag	Inverted	AL	0.52	-	2017	[223]
ITO/ZnO/P3HT:Mn-doped ZnS/MoO_3_/Ag			0.83			
ITO/ZnO/PTBT:PC70BM:InP-ZnS/MoO_3_/Ag	Inverted	AL	10.2	9.1	2018	[224]
**Composites based on Cu_2_S or CuS**						
ITO/ZnO/PTB7-Th:Cu_2_S:PCBM/MoO_3_/Ag	Inverted	AL	8.2	6.96	2019	[225]
ITO/ZnO/PBDB-T:Cu_2_S:ITIC/MoO_3_/Ag	Inverted	AL	9.53	8.24	2019	[226]
ITO/PEDOT:PSS/P3HT:PCBM:CuS/LiF/Al	Regular	AL	5.04	2.98	2019	[98]
**Composites based on CuInS_2_ or PbS_x_Se_1-_**_x_						
ITO/PEDOT:PSS/P3HT:PCBM:CuInS_2_/Al	Regular	AL	2.76	2.44	2011	[227]
ITO/PEDOT:PSS/P3HT:CuInS_2_:graphene/LiF/Al	Regular	AL	1.3	-	2014	[228]
ITO/PEDOT:PSS/PDTPBT:PbS_x_Se_1-x_/PbS_x_Se_1-x_/LiF/Al	Regular	AL	5.5	-	2013	[229]
**Composites based on Si**						
ITO/PEDOT:PSS/P3HT:PCBM:Si/Al	Regular	AL	4.16	-	2011	[232]
ITO/PEDOT:PSS/PTB7:Si/Al	Regular	AL	0.03	-	2012	[234]
ITO/PEDOT:PSS/MDMO-PPV:Si/Al	Regular	AL	0.49	-	2012	[236]
ITO/PEDOT:PSS/P3HT:PCBM:Si/Al	Regular	AL	2.7	2.6	2012	[237]
ITO/PEDOT:PSS/P3HT:PCBM:Si/Al	Regular	AL	3.2	2.8	2012	[101]
ITO/PEDOT:PSS/P3HT:Si/Al	Regular	AL	0.15	-	2013	[238]
ITO/PEDOT:PSS/MEH-PPV:PCBM:Si/Al	Regular	AL	2.28	1.92	2013	[239]
ITO/PEDOT:PSS/P3HT:PCBM:Si/Ca/Al	Regular	AL	3.38	3.01	2013	[240]
ITO/PEDOT:PSS/P3HT:Si/Al	Regular	AL	0.08	-	2014	[174]
ITO/PEDOT:PSS:Si/MEH-PPV:PCBM/Al	Regular	ABL	2.46	2.24	2014	[97]
ITO/PEDOT:PSS/MEH-PPV:PS-Si/Al	Regular	AL	0.03073	0.00008	2015	[241]
ITO/PEDOT:PSS/Si:PTB7/PC71BM/Al	Regular	AL	2.46	-	2016	[231]
FTO/PEDOT:PSS/P3HT:Si/Al	Regular	AL	1.067	0.099	2018	[242]
ITO/ZnO/P3HT:PCBM:p-Si/MoO_3_/Al	Inverted	AL	3.46	2.8	2018	[104]
ITO/ZnO/P3HT:PCBM:n-Si/MoO_3_/Al			3.13			
ITO/PEDOT:PSS/P3HT:Si/Ca/Al	Regular	AL	3.77	-	2019	[243]

## Data Availability

Not applicable.
